# Engineered Xenogeneic Bone Scaffold with IL‐10 Nanodelivery System: Immunomodulation and BMSC Fate Programming for Skull Defect Repair

**DOI:** 10.1002/advs.76971

**Published:** 2026-08-05

**Authors:** Weihao Lv, Lei Zhang, Fei Fei, Zhibin Wu, Xiuquan Wu, Ya‐nan Dou, Yihao Fu, Hao Chang, Yujie Qiang, Huaxia Xie, Ni An, Leiying Chen, Hongchen Zhang, Min‐Wen Zheng, Hongqing Chen, Xia Li, Zhou Fei

**Affiliations:** ^1^ Department of Neurosurgery Xijing Hospital Air Force Medical University (Fourth Military Medical University) Xi'an Shaanxi China; ^2^ Department of Ophthalmology Xijing Hospital Air Force Medical University (Fourth Military Medical University) Xi'an Shaanxi China; ^3^ Department of Radiology Xijing Hospital Air Force Medical University (Fourth Military Medical University) Xi'an Shaanxi China

**Keywords:** immunomodulation, interleukin‐10, mesenchymal stem cells, osteogenesis, skull defect repair, xenogeneic bone scaffold

## Abstract

Skull defect reconstruction remains clinically challenging because current artificial implants lack bioactivity and produce radiological artifacts, whereas autologous bone grafts suffer from unpredictable resorption and infection. Here, we developed a multifunctional composite integrating antigen‐extracted xenogeneic scaffold, IL‐10‐loaded mesoporous polydopamine nanoparticles (MPDA@IL‐10), and autologous bone marrow‐derived mesenchymal stem cells (BMSCs). The composite establishes a temporally phased immunoregulatory axis: nanoparticle‐delivered exogenous IL‐10 compresses the early inflammatory window, and BMSCs then secrete endogenous anti‐inflammatory factors that prevent scaffold resorption and maintain a pro‐regenerative milieu. Beyond immunomodulation, IL‐10 acts as a lineage‐determining cue, activating PI3K‐Akt/Wnt signaling to drive BMSC osteogenic commitment and suppress adipogenic drift at the early differentiation stage. In a canine critical‐sized skull defect model, this strategy markedly enhanced radiographic and histological regeneration. By uniting temporal immune modulation with stem cell fate programming, this approach offers a translatable route to skull repair.

## Graphical abstract

A multifunctional regenerative composite is constructed by 3D‐printing thermosensitive PNIPAM hydrogel embedded with MPDA@IL‐10 nanoparticles and autologous BMSCs onto antigen‐depleted porcine bone matrix for beagle critical‐sized skull defect reconstruction. Sustained IL‐10 delivery regulates macrophage polarization and directs BMSC osteogenic differentiation, sequentially remodeling the immune microenvironment and boosting in vivo skull bone regeneration.

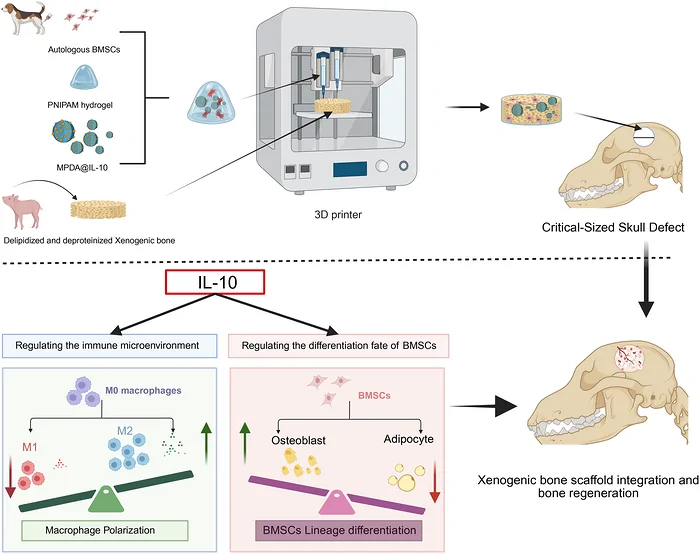

## 1 Introduction

Skull defects from high‐energy trauma, combat injuries, or decompressive craniectomy pose formidable reconstructive challenges. Beyond mechanical compromise, they cause progressive neurological deterioration—disrupted cerebrospinal fluid dynamics, cognitive deficits, and life‐threatening brain herniation—severely compromising quality of life [1, 2, 3, 4]. Craniofacial defects, including cranio‐orbital bone loss, engender similar complications [5, 6]. Current strategies rely mainly on artificial implants (e.g., polyetheretherketone [PEEK] and titanium mesh), which offer good mechanical strength and processability but are bioinert, produce radiological artifacts, and are costly [7]. Autologous bone, the gold standard for its histocompatibility and low immunogenicity, remains prone to unpredictable resorption and infection after flap repositioning, frequently causing reconstructive failure [8]. To achieve regenerative skull reconstruction, we previously developed a preclinical 3D‐bioprinted construct combining autologous bone marrow‐derived mesenchymal stem cells (BMSCs) with autologous bone powder [9]. Although this enhanced osteogenic differentiation, critical barriers persisted: inadequate mechanical support for load‐bearing cranioplasty and central inflammation with fibroblast infiltration that undermines osteogenesis. These challenges underscore the need for a composite system that simultaneously provides structural support, resolves pathological inflammation, and preserves stem cell‐driven regeneration for durable skull repair.

Tissue engineering offers a paradigm for biological skull reconstruction based on seed cells, bioactive factors, and scaffolds [10]. BMSCs are optimal seed cells owing to their self‐renewal and multilineage differentiation potential [11, 12]. Interleukin‐10 (IL‐10) is a pivotal regulator of inflammation and bone metabolic homeostasis; by suppressing pro‐inflammatory cytokines (TNF‐α, IL‐1β, and IL‐6), it attenuates inflammatory bone resorption [13]. In osteoporosis and inflammatory bone diseases, elevated pro‐inflammatory cytokines impair BMSC proliferation, disrupt niche integrity, and ultimately cause bone loss, impaired bone quality, and aberrant adipose accumulation [14, 15]. IL‐10‐deficient mice exhibit osteoporotic phenotypes, impaired bone formation, and delayed fracture healing, confirming its indispensable role in physiological bone metabolism [16, 17]. Collectively, whether by indirectly modulating osteoclast function through inflammation suppression or by directly participating in BMSC osteogenic differentiation, IL‐10 is a promising factor for tissue engineering. However, its short half‐life, proteolytic susceptibility, and risk of systemic immunosuppression necessitate localized, sustained delivery. To address this, mesoporous polydopamine (MPDA) is an ideal nanocarrier, offering a large surface area, uniform mesoporous architecture, and excellent biocompatibility for efficient IL‐10 loading and protection from degradation [18, 19]. Poly(*N*‐isopropylacrylamide) (PNIPAM) thermoresponsive hydrogel serves as a sustained‐release matrix, undergoing rapid sol‐gel transition at physiological temperature to form a three‐dimensional scaffold that immobilizes BMSCs and MPDA@IL‐10 complexes while mimicking native extracellular matrix [20, 21, 22]. Nevertheless, PNIPAM hydrogels have insufficient mechanical strength and osteoconductivity, making them inadequate as standalone materials for load‐bearing skull reconstruction [23].

Xenogeneic bone offers abundant availability, natural trabecular architecture, and excellent osteoconductivity [24]. Porcine skull bone closely approximates the human calvarium in structure and mechanical properties [25, 26]. However, it contains immunogenic α‐Gal epitopes and major histocompatibility complex (MHC) molecules that elicit host rejection [27]. We therefore used combined delipidation‐deproteinization for deep antigen removal while preserving the native three‐dimensional bone matrix and mineral phase. Beforehand, cortical perforation created interconnected macroporous channels through cortical bone while preserving cancellous porosity, enhancing cell accommodation, vascular ingrowth, and nutrient transport [28, 29, 30].

Here, we develop a multifunctional composite of antigen‑depleted xenogeneic bone, MPDA for controlled IL‐10 release, PNIPAM thermosensitive hydrogel, and autologous BMSCs. It establishes sequential immune regulation: exogenous IL‑10 provides rapid early anti‑inflammatory effects, while BMSCs deliver sustained endogenous immunomodulation. Mechanistically, IL‑10 directly programs BMSC fate by activating the PI3K‑Akt/Wnt pathway to promote osteogenesis and inhibit adipogenesis at the early lineage‑determination stage. In a canine critical‑sized skull defect model, the system markedly enhances radiographic and histological regeneration. This work establishes an immuno‑regenerative paradigm that couples temporal immune remodeling with stem cell fate programming, redefining IL‑10 as a dual‑function regulator of immunity and stem cell fate and offering a translatable approach for clinical skull reconstruction.

## 2 Results

### 2.1 Material Characterization of Xenogeneic Bone Scaffold, MPDA Nanoparticles, and PNIPAM Thermosensitive Hydrogel System

After perforation, the Bama mini‐pig skull bone showed regular through‐holes, and scanning electron microscopy (SEM) revealed interconnected porous networks (Figure 1A). GS‐IB4 immunofluorescence and MHC‐I/MHC‐II immunohistochemistry confirmed near‐complete antigen elimination after delipidation and deproteinization (Figure 1B–E). Residual host‐cell DNA is a key determinant of biological‐product safety [31]; residual DNA in the xenograft scaffold was significantly reduced after delipidation and deproteinization (Figure 1F). Transmission electron microscopy shows MPDA as uniform spherical particles with rough mesoporous surfaces (Figure 1G), and dynamic light scattering determines a mean diameter of 136.16±17.22 nm (Figure 1H) with a PDI of 0.18±0.02 and zeta potential of 3.66±1.91 mV; this nanosystem exhibits an IL‐10 loading capacity of 2.09% and encapsulation efficiency of 64.1%. Nitrogen adsorption‐desorption measurements characterized the surface area and pore structure of the mesoporous polydopamine (MPDA), which showed a typical type‐IV isotherm confirming a mesoporous structure. The Brunauer‐Emmett‐Teller specific surface area was calculated to be 24.49 m^2^/g. t‐Plot analysis showed a micropore area of only 2.49 m^2^/g vs. an external surface area of 21.99 m^2^/g, indicating that active sites were predominantly on the external surface and within the mesoporous channels. According to the Barrett‐Joyner‐Halenda model, the average pore diameter calculated from the desorption branch was 27.28 nm, with a pore volume of 0.183 cm^3^/g (Figure 1I,J). The PNIPAM thermosensitive hydrogel was then characterized. Figure 1K shows its chemical structure, and SEM shows a three‐dimensional porous architecture (Figure 1L). The Fourier transform‐infrared (FTIR) spectra of poly (N‐isopropylacrylamide) (PNIPAM) exhibit characteristic absorption peaks: 3282 cm^−^^1^ (N–H stretching, hydrogen‐bonded), 3080 cm^−^^1^ (amide II overtone), 2974/2934 cm^−^^1^ (C–H stretching of isopropyl –CH(CH_3_)_2_), 1628 cm^−^^1^ (amide I, C═O stretching), 1545 cm^−^^1^ (amide II, N–H bending + C–N stretching), 1459 cm^−^^1^ (methyl bending), 1387/1369 cm^−^^1^ (isopropyl fingerprint doublet, umbrella vibration coupling), 1172 cm^−^^1^ (C–N stretching, amide III region), and 506 cm^−^^1^ (low‐frequency skeletal vibration). The absence of C═C stretching in the 1634–1700 cm^−^^1^ region confirms complete vinyl consumption and successful polymerization (Figure 1M). Below the lower critical solution temperature (LCST), cooperative hydration of bound water molecules forms “hydrogen‐bond bridges” around hydrophobic isopropyl groups, stabilizing chains in an extended, soluble coil conformation. Above the LCST, cooperative dehydration disrupts these bridges; the mixing free energy becomes positive, driving hydrophobic collapse into compact globules and interchain hydrogen bonding (N─H···O═C) that physically cross‐link the network. A general diagram of the phase transition of PNIPAM and rheological analysis confirms thermosensitive phase transition at 32°C–35°C (Figure 1N,O). Degradation curves indicate >70% mass retention at 7 days (Figure 1P). Importantly, compared with free MPDA@IL‐10, the PNIPAM@MPDA@IL‐10 composite achieved ∼35% cumulative release at 7 days (Figure 1Q), confirming controlled release. The released IL‐10 remained bioactive: 7‐day sustained release reduced the pro‐inflammatory macrophage phenotype and macrophage inflammatory‐factor expression (Figure S1).

**FIGURE 1:**
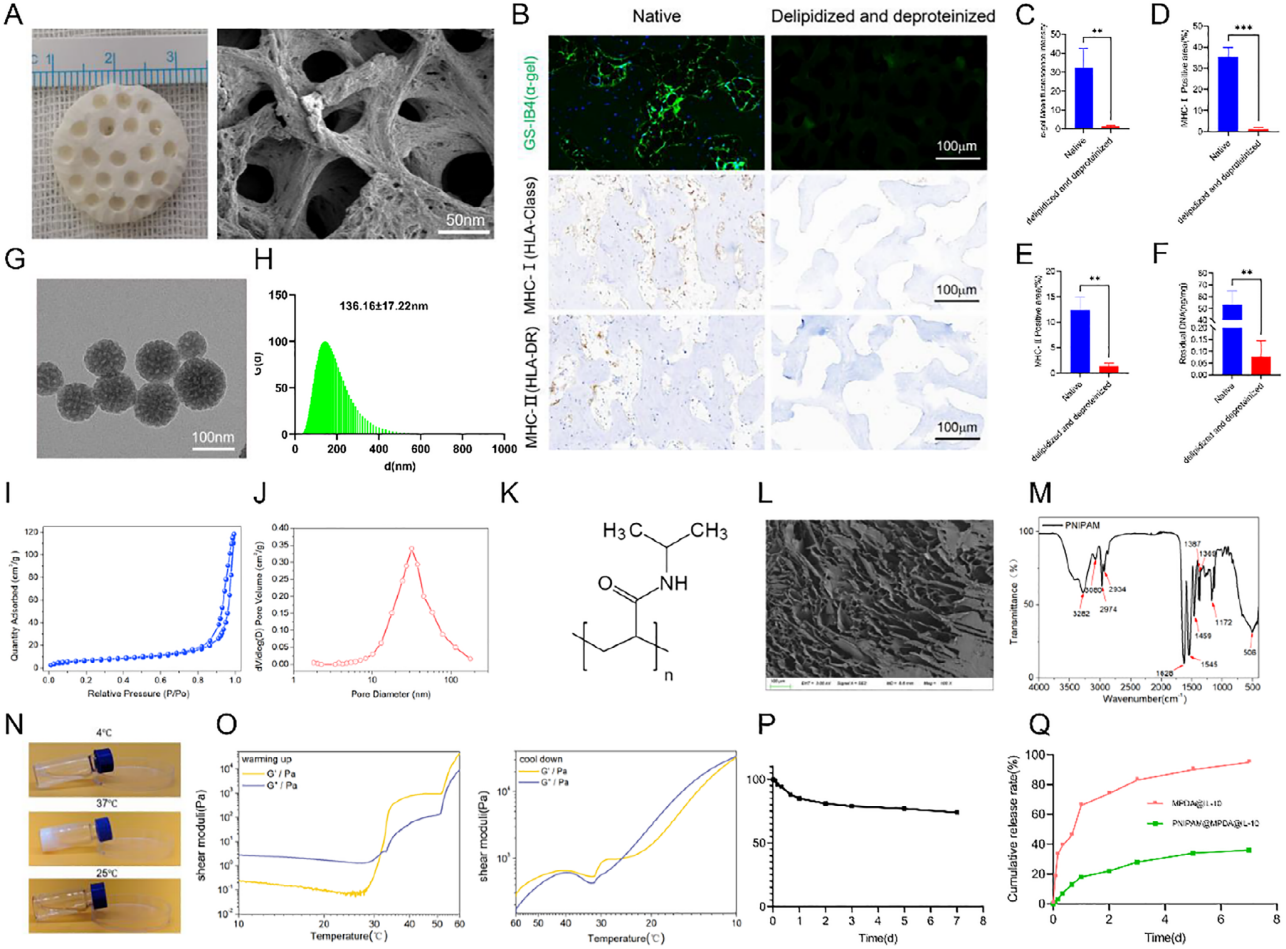
Material characterization of xenogeneic bone scaffold, MPDA nanoparticles, and PNIPAM thermosensitive hydrogel system. (A) Gross appearance (left) and SEM image (right) of porogenated Bama mini‐pig skull bone scaffold. (B) GS‐IB4 immunofluorescence staining (top row), MHC‐I immunohistochemical staining (middle row), and MHC‐II immunohistochemical staining (bottom row) of xenogeneic bone before and after treatment: left column shows Native bone, right column shows Delipidized and deproteinized bone. (C‐E) Quantitative analysis of α‐gel, MHC‐I, and MHC‐II. (F) Quantification of residual DNA. (G) TEM image of MPDA nanoparticles. (H) DLS size distribution of MPDA nanoparticles, with a mean diameter of 136.16±17.22 nm. (I) Nitrogen sorption isotherm. (J) Pore size distribution. (K) Chemical structure of PNIPAM. (L) SEM image of PNIPAM thermosensitive hydrogel. (M) FTIR spectrum of PNIPAM. (N) General diagram of phase transition of PNIPAM hydrogel with temperature change. (O) Rheological temperature sweep curve of PNIPAM thermosensitive hydrogel: black curve indicates storage modulus (G'), red curve indicates loss modulus (G''). (P) In vitro degradation curve of PNIPAM thermosensitive hydrogel. (Q) In vitro IL‐10 release curves: red curve indicates MPDA@IL‐10 group, green curve indicates PNIPAM@MPDA@IL‐10 group. For panel C‐F: ^**^ *p*< 0.01 by Student's *t*‐test. Data are presented as the mean ± SD, *n* = 3/group.

### 2.2 Characterization of Canine BMSCs and Biocompatibility Evaluation

Having verified the structural and sustained‐release properties of the composites, we isolated and identified canine BMSCs and evaluated the biocompatibility of all materials before in vivo application. Primary canine BMSCs exhibited typical fibroblast‐like morphology with a swirling pattern (Figure 2A). Flow cytometry confirmed high expression of the mesenchymal markers CD90 (99.51%) and CD44 (99.43%) and negligible expression of the hematopoietic marker CD34 (1.96%) and monocyte marker CD14 (0.97%), indicating high purity and mesenchymal identity (Figure 2B). Figure 2C demonstrated the trilineage differentiation potential of canine BMSCs—positive alkaline phosphatase (ALP) staining and alizarin red staining (ARS) showing calcium nodule formation after osteogenic induction, positive alcian blue staining after chondrogenic induction, and lipid‐droplet formation by oil red O staining after adipogenic induction. Biocompatibility is shown in Figure 2D–K: live/dead staining showed no obvious dead cells in the xenogeneic bone, PNIPAM hydrogel, or PNIPAM@MPDA@IL‐10 composite extract groups vs. the control, with no difference in viability (Figure 2D,E); EdU proliferation and cell‐cycle assays confirmed that the extracts did not affect BMSC proliferation (Figure 2F–I); and Transwell assays showed no effect on migration (Figure 2J,K). Notably, SEM (Figure 2L) showed canine BMSCs adhering extensively to the delipidized, deproteinized xenogeneic bone with robust spreading and abundant pseudopodia, confirming excellent cytocompatibility for subsequent composite construction.

**FIGURE 2:**
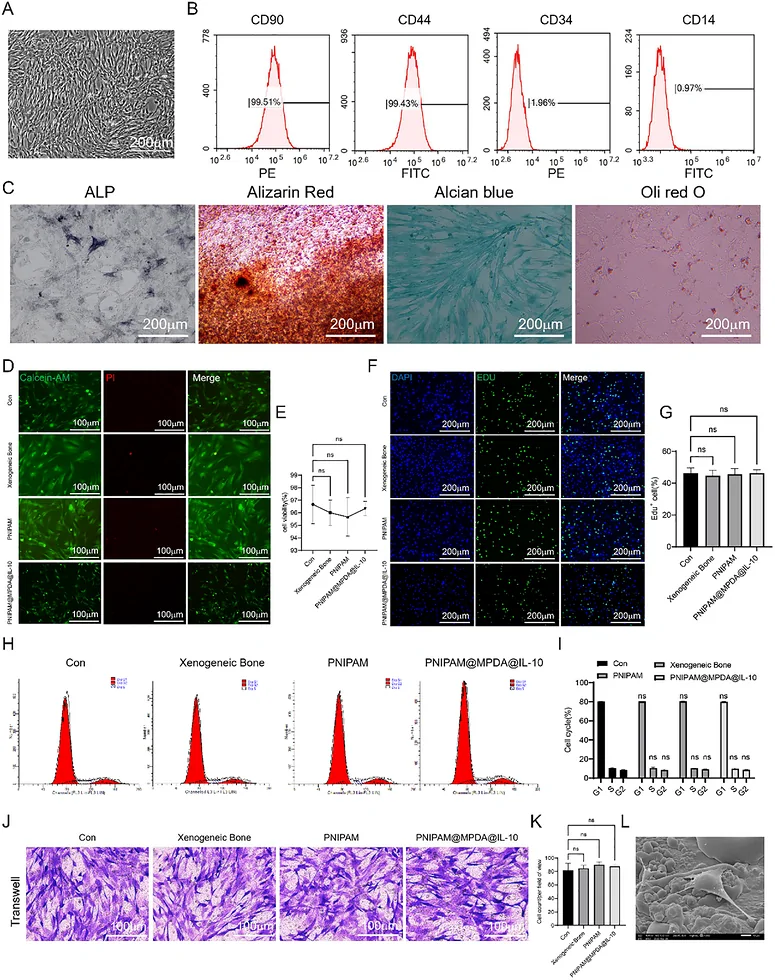
Characterization of canine BMSCs and biocompatibility evaluation. (A) Light microscopy image of canine BMSCs. (B) Flow cytometry detection of surface markers in canine BMSCs. (C) Trilineage differentiation staining images of canine BMSCs: ALP staining, ARS staining, alcian blue staining, and oil red O staining. (D) Live/dead cell staining (Calcein‐AM/PI) images of each group (Con: control; Xenogeneic Bone: xenogeneic bone extract; PNIPAM: thermosensitive hydrogel extract; PNIPAM@MPDA@IL‐10: composite extract). (E) Quantitative statistical graph of live/dead cell staining. (F) EdU cell proliferation staining images of each group. (G) Quantitative statistical graph of EdU‐positive cell proportion. (H) Flow cytometry detection results of cell cycle in each group. (I) Quantitative statistical graph of cell cycle distribution in each group. (J) Transwell cell invasion assay images of each group. (K) Quantitative statistical graph of cell numbers across membranes. (L) SEM image of canine BMSCs attached to the surface of delipidized and deproteinized xenogeneic bone. For panel E, G, I, K: ns, no significance by one‐way ANOVA analysis. Data are presented as mean ± SD. *n* = 3/group.

### 2.3 BMSCs + IL‐10 Composite System Significantly Promotes Radiographic Bone Healing in a Critical‐Sized Skull Defect

CT monitoring from 1 to 6 months postoperatively revealed significant differences in bone healing among groups (Figure 3A). The Blank group showed persistent non‐union. The Xenograft‐alone group showed progressive scaffold resorption, with marked volume loss by 6 months and no effective repair. The IL‐10 group also showed obvious resorption with scaffold displacement and poor healing. The BMSCs group showed attenuated resorption and improved healing vs. these two groups. In contrast, the BMSCs + IL‐10 group showed a distinct pattern: an intact scaffold without significant resorption and the most prominent osseointegration. Micro‐CT reconstruction at 6 months (Figure 3B) confirmed these patterns: the blank group had almost no new bone; the Xenograft‐alone group, a destroyed scaffold; the IL‐10 group, an incomplete scaffold with local resorption and minimal new bone; the BMSCs group, increased but uneven new bone; and the BMSCs + IL‐10 group, extensive new bone (yellow) surrounding and growing into the scaffold (gray) with preserved integrity. Quantitative analysis (Figure 3C–F) showed that both the xenogeneic bone scaffold and new bone in the BMSCs + IL‐10 group exhibited significantly higher bone mineral density (BMD) and bone volume fraction (BV/TV) than all other groups. Histology at 6 months (Figure 3G) revealed the microstructural basis of repair. The Blank group contained fibrous connective tissue without mature trabeculae; the xenograft and IL‐10 groups showed scaffold fragments, inflammatory infiltration, and disorganized collagen with sparse trabeculae; and the BMSCs group showed increased collagen and immature trabeculae. In contrast, the BMSCs + IL‐10 group showed dense lamellar bone, well‐aligned mature collagen, tight bone‐scaffold integration, and nascent marrow cavities with minimal inflammation, consistent with imaging. Importantly, this strategy suppressed xenograft resorption and achieved structural bone healing at the defect.

**FIGURE 3:**
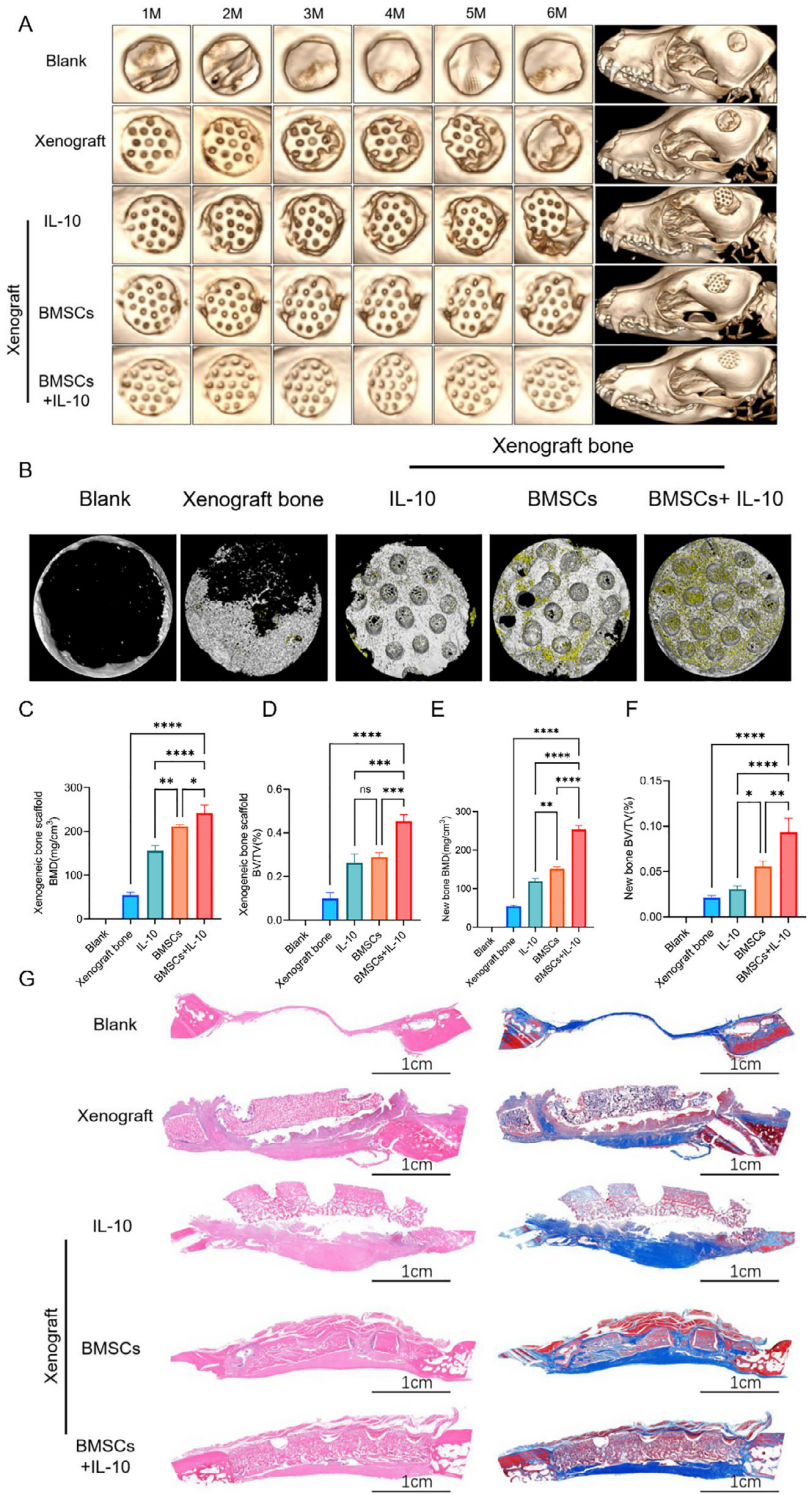
BMSCs + IL‐10 composite system promotes repair of critical‐size skull defect in beagles. (A) Regular CT images of skull defect areas in each group (Blank, Xenograft, IL‐10, BMSCs, BMSCs + IL‐10) at 1–6 months postoperatively. (B) Micro‐CT three‐dimensional reconstruction images at 6 months postoperatively: gray indicates implanted xenograft scaffold, yellow indicates new bone. (C) Quantitative analysis of xenograft scaffold BMD at 6 months post‐surgery. (D) Quantitative analysis of xenogeneic bone scaffold BV/TV at 6 months post‐surgery. (E) Quantitative analysis of new bone BMD at 6 months post‐surgery. (F) Quantitative analysis of new bone BV/TV at 6 months post‐surgery. (G) Representative H&E (left) and Masson's trichrome (right) staining of skull defect sections at 6 months post‐surgery. For panel C‐F: ns, no significance; ^*^ *p*< 0.05; ^****^ *p*< 0.0001 by one‐way ANOVA analysis. Data are presented as the mean ± SD, *n* = 3/group.

### 2.4 Sequential Immune Remodeling Creates a Pro‐Regenerative Microenvironment

The prognosis of large‐segment bone defect repair depends heavily on the immune‐inflammatory status of the defect [32], and after xenogeneic scaffold implantation, both innate and adaptive immunity determine scaffold retention and osseointegration. We first examined adaptive immune rejection: flow cytometry (Figure 4A) showed no significant differences in CD4^+^ helper T cells, CD8^+^ cytotoxic T cells, or the CD4^+^/CD8^+^ ratio among groups at any postoperative time point, indicating that the delipidated, deproteinized scaffold evaded T cell‐mediated rejection and setting the stage for examining innate immune regulation.

**FIGURE 4:**
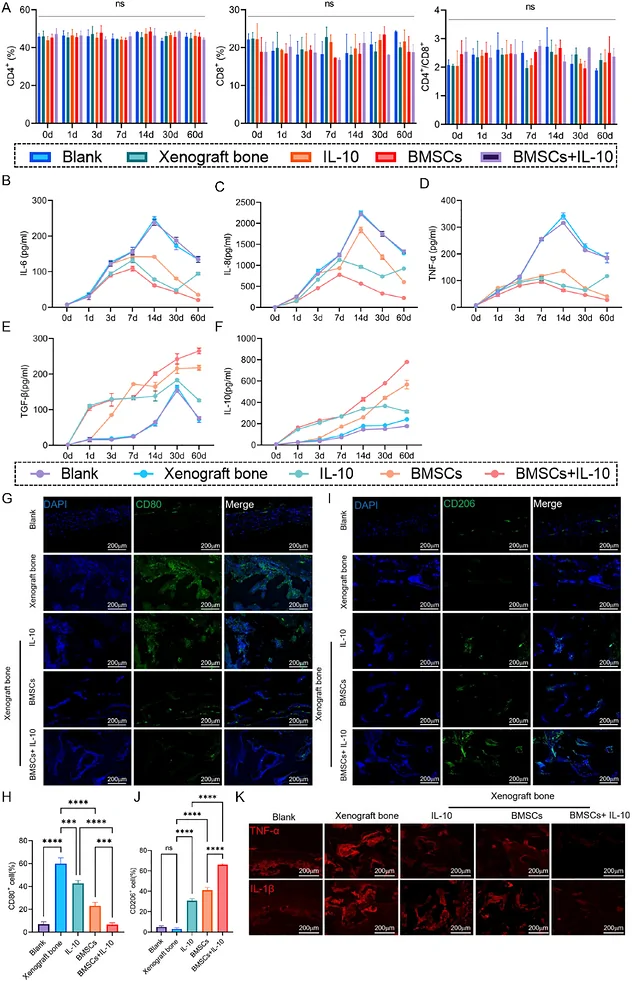
Immune microenvironment regulation and inflammatory cytokine changes in critical‐size skull defect repair. (A) Flow cytometric analysis of CD4^+^, CD8^+^ T cell proportions and CD4^+^/CD8^+^ ratio at indicated time points post‐surgery. (B–F) Dynamic detection of pro‐inflammatory cytokines (IL‐6, IL‐8, TNF‐α) and anti‐inflammatory cytokines (TGF‐β, IL‐10) in peripheral serum. (G,I) Representative immunofluorescence images of CD80 (G) and CD206 (I) expression in defect areas at 6 months post‐surgery. (H,J) Quantitative analysis of CD80^+^ (H) and CD206^+^ (J) cell proportions. (K) Immunofluorescence staining of local TNF‐α and IL‐1β expression in defect areas. For panel H‐J: ns, no significance; ^****^ *p*< 0.0001 by one‐way ANOVA analysis. Data are presented as mean ± SD, *n* = 3/group.

Serum cytokine monitoring revealed material‐dependent differences in the “inflammatory‐to‐reparative transition” time window (Figure 4B–F). The Xenograft group showed a typical post‐traumatic response: pro‐inflammatory IL‐6, IL‐8, and TNF‐α peaked at 7–14 days and resolved slowly, reflecting immune recognition of the xenogeneic material and a delayed M1‐to‐M2 transition. Through nanoparticle‐mediated sustained release, the IL‐10 group suppressed TNF‐α and IL‐6 peaks as early as 3–7 days, reducing peak duration by ∼50% for rapid early anti‐inflammatory effects; however, this elevation was transient and declined after 14 days as exogenous IL‐10 waned. The BMSCs group showed a moderate response: pro‐inflammatory cytokines (IL‐6, IL‐8, and TNF‐α) peaked at 7–14 days, below the xenograft group but above the BMSCs + IL‐10 group, while anti‐inflammatory factors (IL‐10 and TGF‐β) rose gradually from 7 days. Thus, BMSCs alone partially mitigate inflammation via paracrine effects but with delayed onset and less sustained capacity than the combination. Notably, the BMSCs + IL‐10 group showed optimal temporal complementarity: during days 14–30, as exogenous IL‐10 became depleted, BMSCs continuously secreted endogenous IL‐10 and TGF‐β [33, 34, 35], creating an “exogenous initiation‐endogenous maintenance” transition. This yielded the highest anti‐inflammatory and lowest pro‐inflammatory factor levels of all groups.

Histological analysis of the local immune microenvironment at 6 months strengthened these conclusions (Figure 4G–J): CD80^+^ pro‐inflammatory M1 macrophages were significantly reduced in the BMSCs + IL‐10 group, while CD206^+^ anti‐inflammatory M2 macrophages were markedly increased, reflecting a shift of the local microenvironment from pro‐inflammatory to pro‐regenerative. Immunofluorescence for local pro‐inflammatory cytokines (TNF‐α and IL‐1β; Figure 4K) showed both were significantly suppressed in the BMSCs + IL‐10 group, confirming that this strategy creates a low‐inflammatory, pro‐regenerative microenvironment for scaffold osseointegration and new‐bone maturation by regulating macrophage polarization and inhibiting local chronic inflammation.

### 2.5 BMSCs + IL‐10 Composite System Significantly Promotes Histological Bone Regeneration in a Critical‐Sized Skull Defect

Having shown that the BMSCs + IL‐10 system regulates innate immunity and builds a low‐inflammatory, pro‐repair microenvironment, we next investigated how it translates these immune effects into direct bone regeneration in the critical‐size skull defect. Immunofluorescence and quantification of osteogenic markers in the defect at 6 months showed that osteocalcin (OCN), Runt‐related transcription factor 2 (RUNX2), osteopontin (OPN), and type I collagen α1 chain (Col1a1) were extremely low in the blank and xenograft groups, indicating almost stagnant regeneration. These markers were slightly higher in the IL‐10‐alone and BMSCs‐alone groups, but the positive‐cell fraction remained significantly below the BMSCs + IL‐10 group, reflecting limited osteogenic differentiation. In contrast, OCN, RUNX2, OPN, and Col1a1 signals were significantly enhanced in the BMSCs + IL‐10 group, with a positive‐cell fraction significantly higher than all other groups (Figure 5A–H). Thus, the BMSCs–IL‐10 synergy resolves the inflammatory rejection from xenogeneic scaffold implantation and improves the quality and efficiency of regeneration by enhancing osteoblast differentiation and matrix mineralization, ultimately healing the critical‐size skull defect.

**FIGURE 5:**
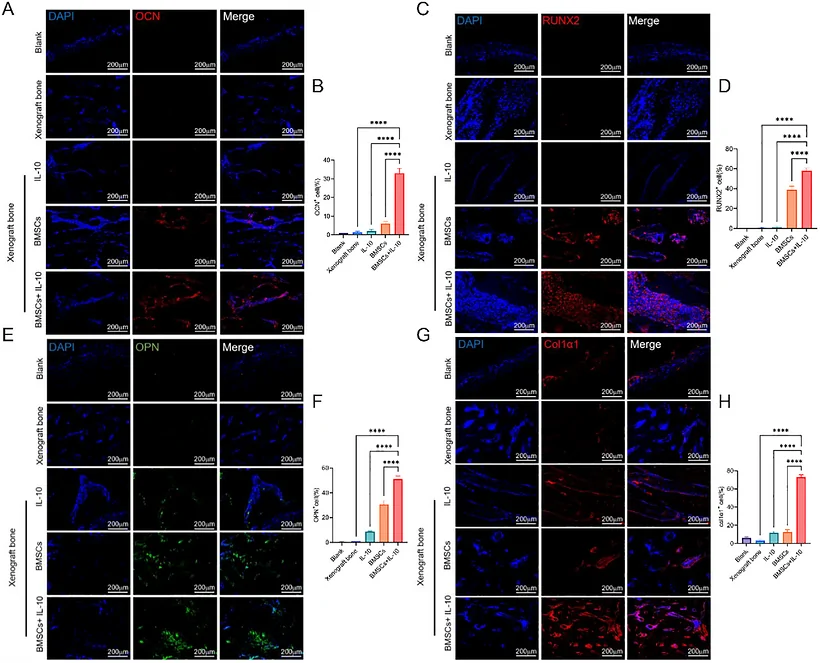
BMSCs–IL‑10 system promotes histological bone regeneration in vivo. (A,C,E,G) Representative immunofluorescence images of OCN (A), RUNX2 (C), OPN (E), and Col1a1 (G) expression in the defect area at 6 months post‐surgery. (B,D,F,H) Quantitative analysis of the proportion of OCN^+^ (B), RUNX2^+^ (D), OPN^+^ (F), and Col1a1^+^ (H) positive cells. For panels B, D, F, H: ^****^ *p*<0.0001 by one‐way ANOVA analysis. Data are presented as mean ± SD, *n* = 3/group.

### 2.6 IL‐10 Programs BMSC Fate by Promoting Osteogenesis and Inhibiting Adipogenesis

As common progenitors of osteoblasts and adipocytes, BMSCs maintain a balance between osteogenic and adipogenic differentiation that is tightly regulated by multiple signaling pathways [36]. To elucidate IL‐10's direct effect on BMSC fate, we used in vitro differentiation and a nude‐mouse subcutaneous transplantation model to test its impact on the bone‐lipid balance at the cellular and animal levels. In vitro, under osteogenic (OSTE) induction, 10 ng/mL IL‐10 significantly enhanced ALP and ARS staining, with both ALP and mineralized‐nodule area significantly above the control (Figure 6A–C). Under adipogenic (ADI) induction, Oil Red O staining and lipid‐droplet area were markedly reduced by IL‐10 (Figure 6D,E), indicating that IL‐10 directly promotes osteogenic differentiation and inhibits adipogenic differentiation. The nude‐mouse model confirmed these effects (Figure 6F): after 5 weeks, X‐ray showed higher graft bone density in the IL‐10 + BMSCs group than the BMSCs‐alone group, and H&E showed more osteoid and mature bone lacunae in the IL‐10 + BMSCs group, whereas the BMSCs group was dominated by adipose tissue. Immunofluorescence confirmed that, vs. the BMSCs group, adipogenic markers (PLIN1, ADIPOQ) were significantly reduced (Figure 6G–I) and osteogenic markers (OPN, RUNX2) significantly increased (Figure 6J–L) in the IL‐10 + BMSCs group.

**FIGURE 6:**
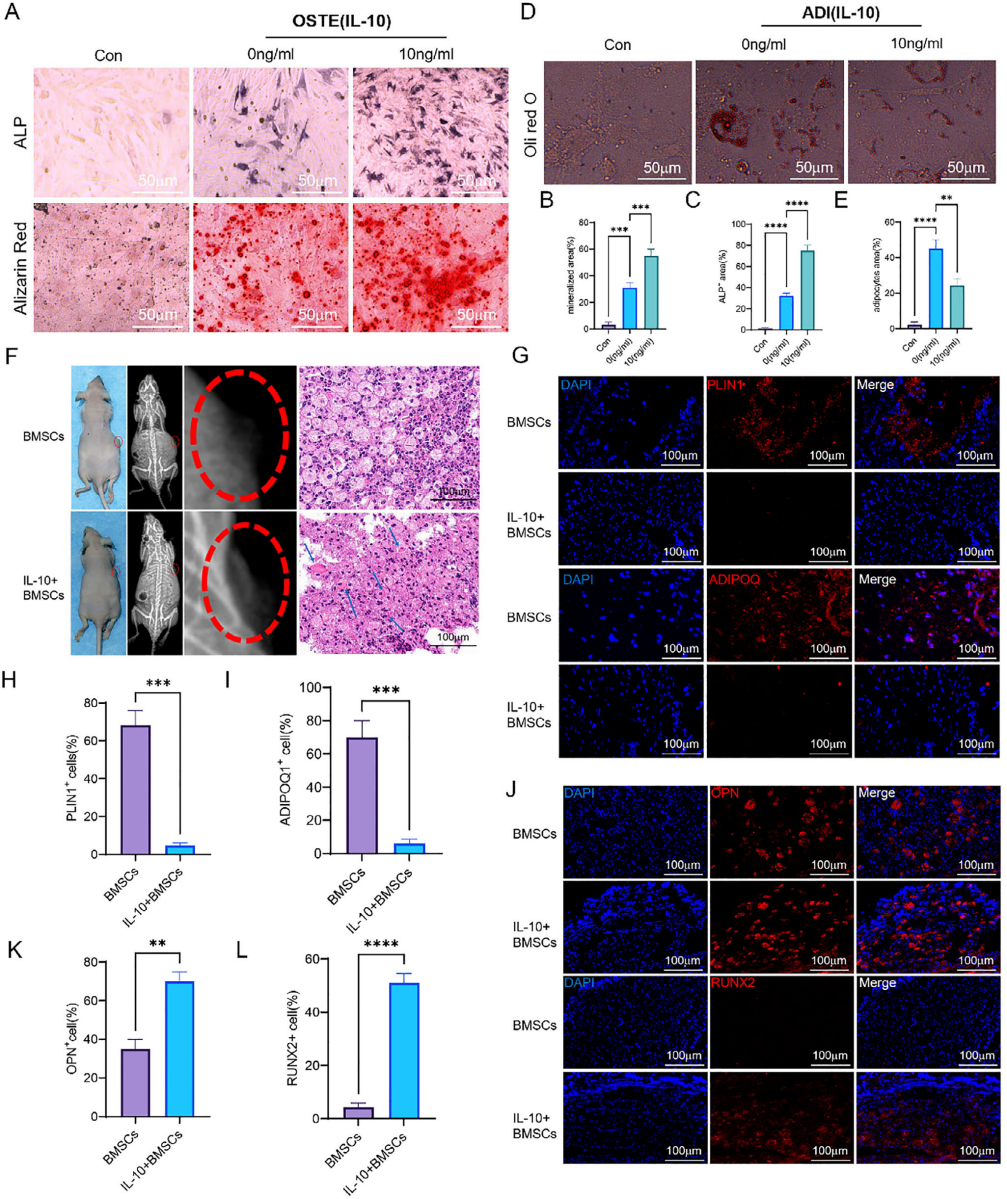
IL‐10 regulates BMSCs differentiation fate by promoting osteogenesis and inhibiting adipogenesis. (A) Representative images of ALP and Alizarin Red S staining of BMSCs under osteogenic differentiation with 0 or 10 ng/mL IL‐10. (B) Quantitative analysis of mineralized nodules area in (A). (C) Quantitative analysis of ALP area in (A). (D) Representative images of Oil Red O staining of BMSCs under adipogenic differentiation with 0 or 10 ng/mL IL‐10. (E) Quantitative analysis of adipocyte area in (D). (F) Representative in vivo x‐ray and H&E staining images of nude mouse heterotopic bone formation (left: gross view; middle: x‐ray view; right: H&E staining). (G) Representative immunofluorescence staining of adipogenic markers PLIN1 and ADIPOQ. (H) Quantitative analysis of PLIN1^+^ cell proportion in (G). (I) Quantitative analysis of ADIPOQ^+^ cell proportion in (G). (J) Representative immunofluorescence staining of osteogenic markers OPN and RUNX2. (K) Quantitative analysis of OPN^+^ cell proportion in (J). (L) Quantitative analysis of RUNX2^+^ cell proportion in (J). For panel B‐E, H‐L: ^**^ *p*<0.01, ^***^ *p*<0.001, ^****^ *p*<0.0001 by one‐way ANOVA analysis or Student's *t*‐test. Data are presented as mean ± SD, *n* = 3/group.

### 2.7 IL‐10 Controls BMSC Lineage Commitment at the Early Fate‐Determination Stage

BMSC differentiation comprises three stages—early commitment, lineage‐specific differentiation, and functional maturation—of which early commitment is the core node determining cell fate [37]. To further elucidate how IL‐10 regulates BMSC fate, we focused on this stage, evaluating IL‐10's dynamic effects throughout osteogenic/adipogenic induction and its short‐term preconditioning effect. Time‐course experiments (Figure 7A–E) showed that under OSTE, 7‐day IL‐10 treatment time‐dependently enhanced ALP activity and mineralized‐nodule formation, with both ALP‐positive and mineralized areas much higher than control. Under ADI, IL‐10 markedly inhibited Oil Red O‐positive lipid droplets, indicating that IL‐10 directs BMSC fate as early as the commitment stage. Short‐term preconditioning (Figure 7F–J) confirmed that just 2 days of IL‐10 pretreatment (IL‐10(2d)) produced durable effects over the subsequent 7‐day induction: vs. control, the preconditioned group showed significantly higher ALP and mineralized‐nodule area under OSTE and significantly lower lipid‐droplet area under ADI. Collectively, IL‐10 promotes osteogenesis and inhibits adipogenesis throughout differentiation and, by acting rapidly at early commitment, strengthens directed osteogenic potential, providing a cellular basis for subsequent bone regeneration.

**FIGURE 7:**
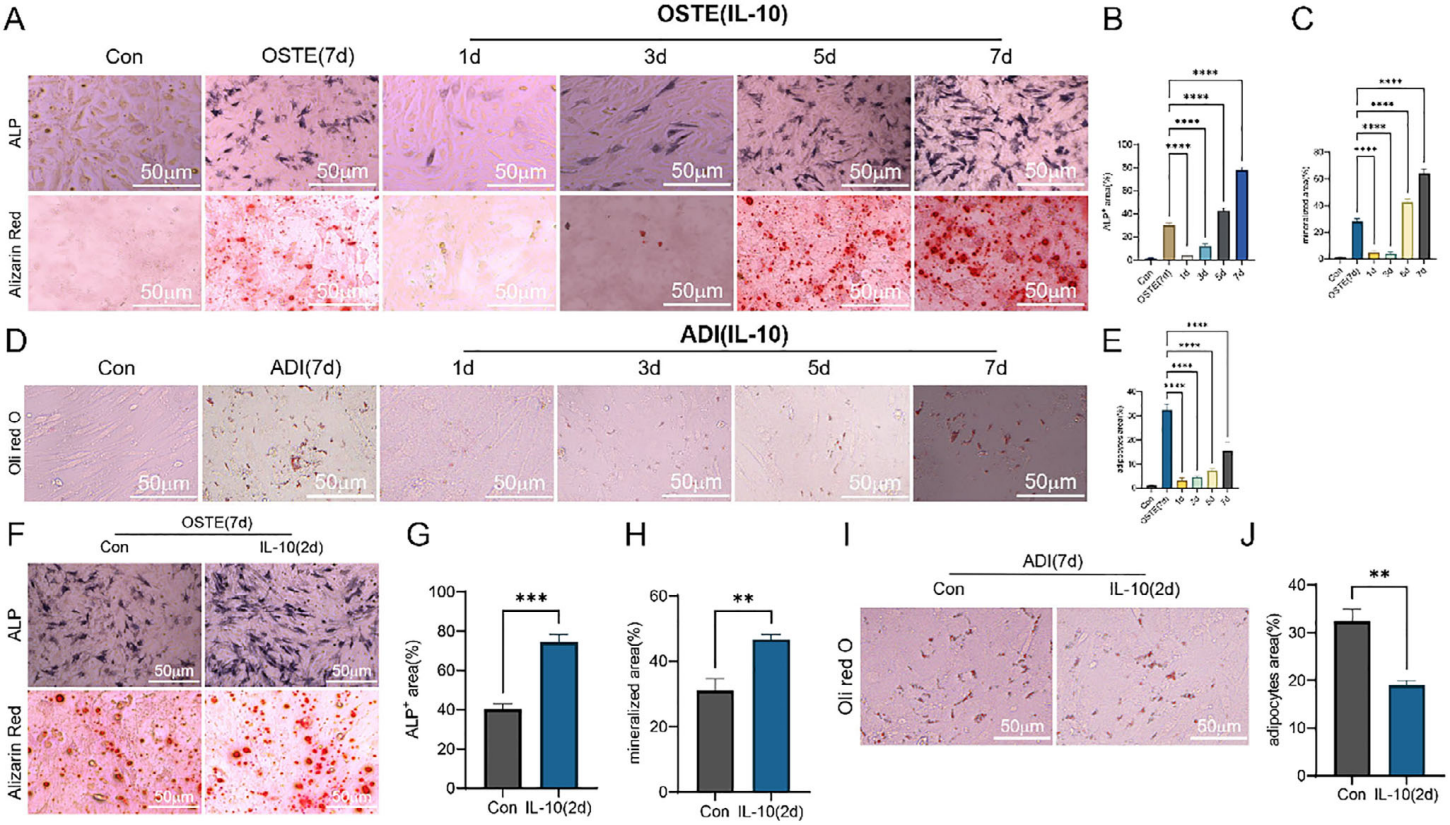
IL‐10 regulates BMSC fate at the early lineage‐commitment stage. (A) Representative ALP and Alizarin Red S staining images of BMSCs during OSTE with IL‐10 treatment at 1, 3, 5, and 7 days. Con: control group; OSTE(7d): osteogenic differentiation for 7 days without IL‐10. (B) Quantitative analysis of mineralized nodule area from (A). (C) Quantitative analysis of ALP area from (A). (D) Representative Oil Red O staining images of BMSCs during ADI with IL‐10 treatment at 1, 3, 5, and 7 days. ADI(7d): adipogenic differentiation for 7 days without IL‐10. (E) Quantitative analysis of adipocyte area from (D). (F) Representative ALP and Alizarin Red S staining images of BMSCs after 2 days of IL‐10 pretreatment followed by 7 days of osteogenic differentiation. (G) Quantitative analysis of ALP area from (F). (H) Quantitative analysis of mineralized nodule area from (F). (I) Representative Oil Red O staining images of BMSCs after 2 days of IL‐10 pretreatment followed by 7 days of adipogenic differentiation. (J) Quantitative analysis of adipocyte area from (I). For panel B‐E, G, H, J: ^**^ *p*<0.01, ^***^ *p*<0.001, ^****^ *p*<0.0001 by one‐way ANOVA analysis or Student's *t*‐test. Data are presented as mean ± SD, *n* = 3/group.

### 2.8 Transcriptomic Profiling Reveals IL‑10–Mediated Lineage Reprogramming

To explore how IL‑10 regulates BMSC fate, we performed RNA‑seq on BMSCs cultured for 5 days in culture medium (CM), ADI, or OSTE medium, with or without 10 ng/mL IL‑10. Heatmaps of differentially expressed genes (Figure 8A–C) showed distinct clustering of IL‑10‑treated vs. control groups under all three conditions, and volcano plots (Figure 8D–F) showed extensive up‐ and downregulation, globally altering the BMSC transcriptome. For these differentially expressed genes (DEGs), Gene Ontology (GO) enrichment (Figure 8G–I) showed that, under all three conditions, IL‐10 significantly enriched osteogenic processes (e.g., “bone mineralization,” “bone development,” and “collagen fibril organization”) while suppressing adipogenic pathways (e.g., “fatty acid biosynthetic process,” “lipid‐droplet formation,” “fat cell differentiation”). qPCR (Figure 8J–L) confirmed these trends: under CM, ADI, and OSTE, IL‐10 significantly upregulated osteogenic transcription factors (OCN, RUNX2, SP7) and inhibited adipogenic genes (CEBPA, Fabp4, PPARγ). Western blot (Figure 8M–R) confirmed this at the protein level: IL‐10 markedly increased osteogenic proteins (ALP, RUNX2) and reduced adipogenic proteins (PPARγ, ADIPOQ). Collectively, these transcriptomic and molecular data show that IL‐10 remodels lineage‐specific gene expression—activating the osteogenic program and suppressing the adipogenic program—providing mechanistic support for its use in bone regeneration.

**FIGURE 8:**
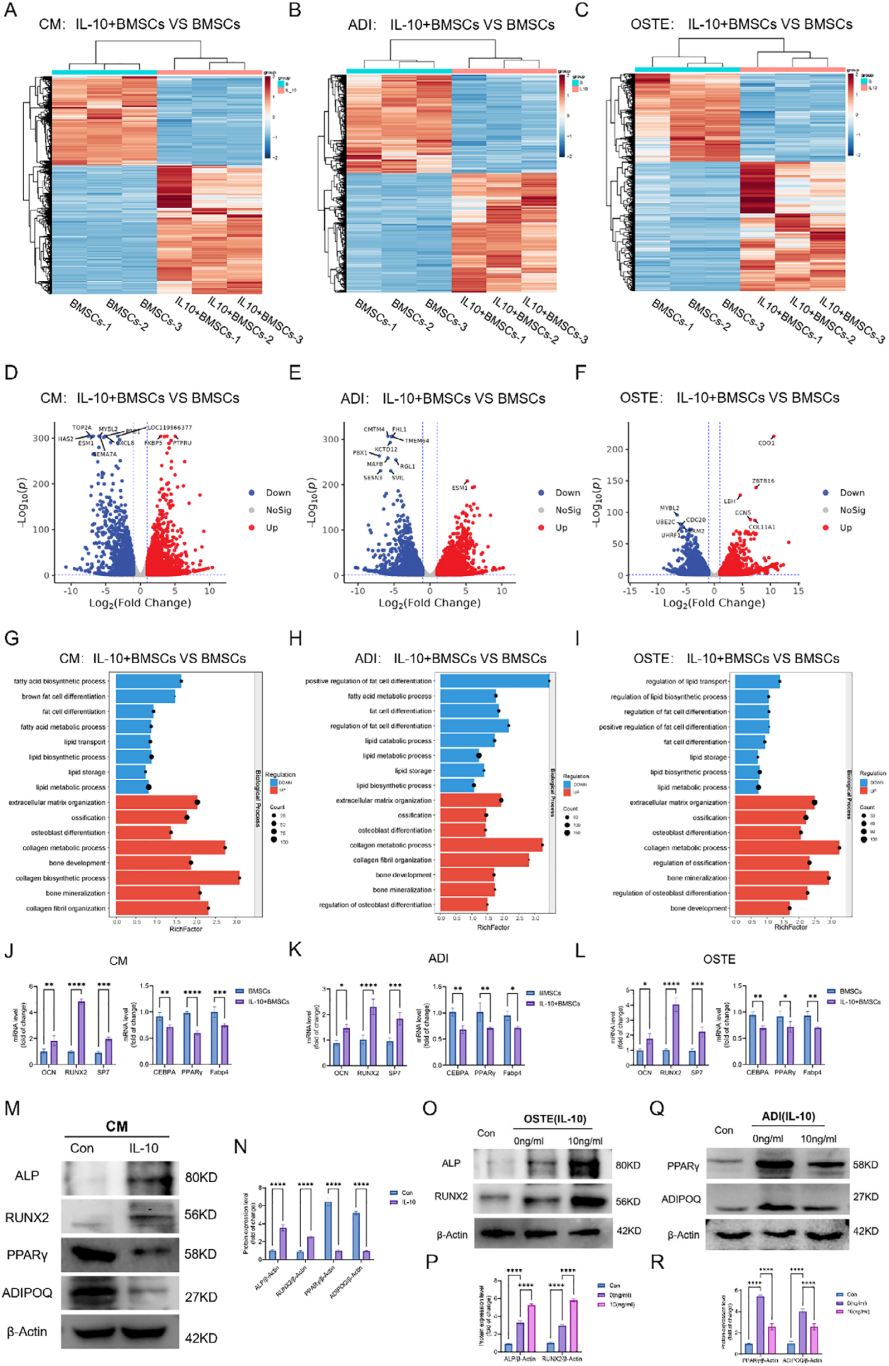
Transcriptomic analysis reveals IL‐10–mediated lineage‐specific gene reprogramming. (A–C) Heatmaps of differentially expressed genes (DEGs) between IL‐10 + BMSCs and BMSCs groups in CM (A), ADI (B), and OSTE (C) conditions. (D–F) Volcano plots of DEGs in CM (D), ADI (E), and OSTE (F) conditions (red: upregulated; blue: downregulated). (G–I) GO enrichment analysis of DEGs in CM (G), ADI (H), and OSTE (I) conditions. (J–L) qPCR validation of osteogenic and adipogenic marker genes in CM (J), ADI (K), and OSTE (L) conditions. (M, O, Q) Western blot analysis of osteogenic and adipogenic proteins in CM (M), OSTE (O), and ADI (Q) conditions. (N, P, R) Quantitative analysis of protein expression levels in CM (N), OSTE (P), and ADI (R) conditions. For panel J‐L, N, P, R: ^*^ *p*<0.05, ^**^ *p*<0.01, ^***^ *p*<0.001, ^****^ *p*<0.0001 by one‐way ANOVA analysis or Student's *t*‐test. Data are presented as mean ± SD, *n* = 3/group.

### 2.9 IL‐10 Regulates BMSCs Differentiation Fate via Co‐Modulated Environmental Information Processing Pathways

To dissect the signaling underlying IL‐10‐mediated BMSC differentiation, we performed KEGG pathway enrichment on DEGs under CM, OSTE, and ADI conditions, focusing on environmental information processing pathways. IL‐10‐activated pathways were culture‐condition‐dependent: under CM, the TNF, PI3K‐Akt, and cytokine‐cytokine receptor interaction pathways were significantly enriched; under ADI, the TNF, PI3K‐Akt, and mTOR pathways predominated; and under OSTE, the TNF, TGF‐β, and PI3K‐Akt pathways were core (Figure 9A–C). Intersection analysis across the three conditions identified 88 commonly upregulated and 12 commonly downregulated genes (100 co‐regulated DEGs; Figure 9D,E). KEGG enrichment of these 100 DEGs revealed three core pathways: ECM‐receptor interaction, PI3K‐Akt, and Wnt signaling (Figure 9F). All are established regulators of BMSC osteogenic and adipogenic differentiation, suggesting that IL‐10 directs BMSC fate by coordinately regulating these conserved pathways.

**FIGURE 9:**
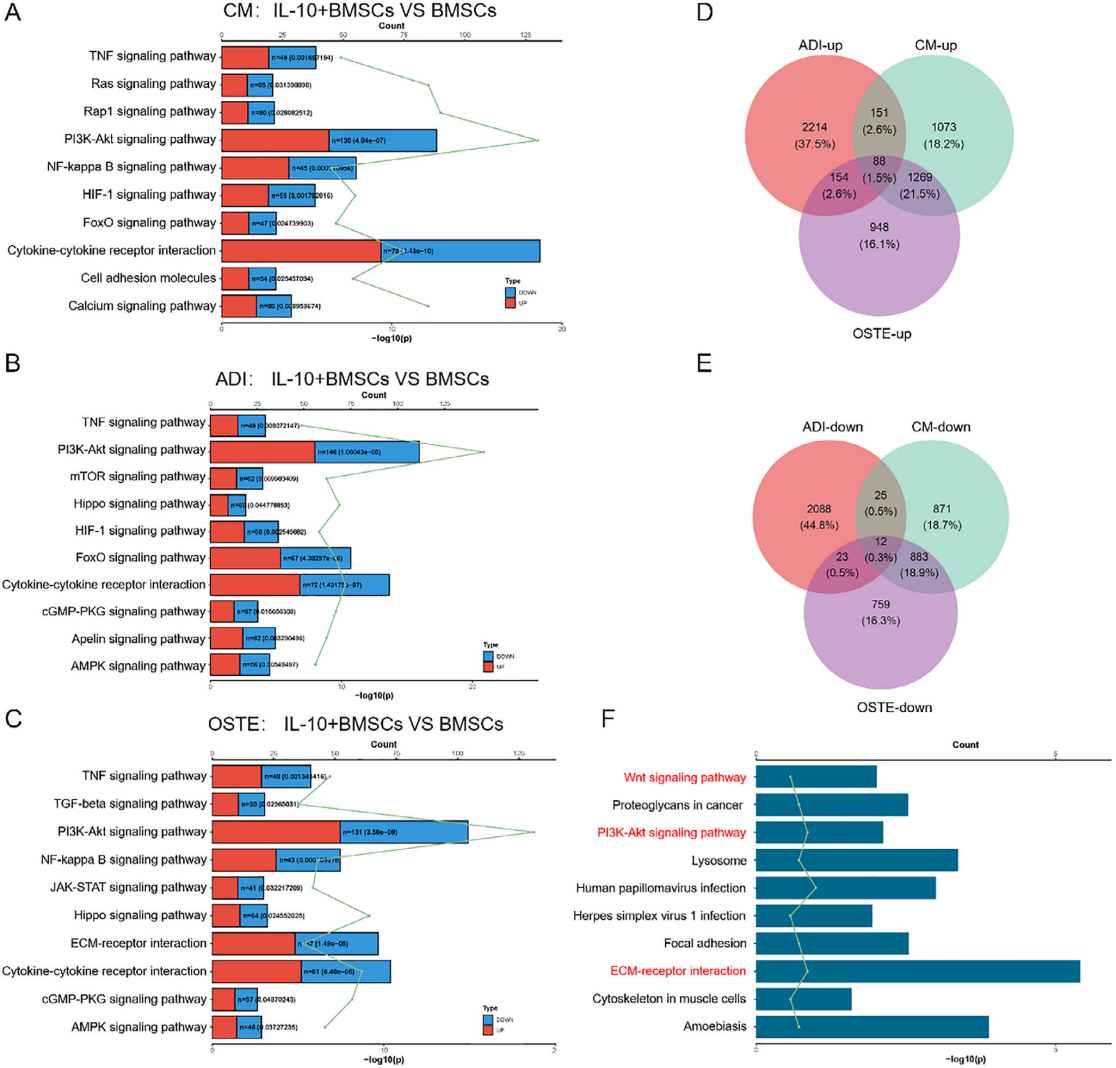
KEGG enrichment analysis reveals co‐regulated environmental information processing pathways underlying IL‐10‐mediated BMSCs differentiation. (A–C) KEGG pathway enrichment analysis of DEGs under CM (A), ADI (B), and OSTE (C) conditions (focused on environmental information processing pathways). (D, E) Venn diagrams of upregulated (D) and downregulated (E) DEGs across three culture conditions, identifying 88 co‐upregulated and 12 co‐downregulated genes. (F) KEGG enrichment analysis of 100 co‐regulated DEGs, highlighting three core pathways: ECM‐receptor interaction, PI3K‐Akt signaling pathway, and Wnt signaling pathway.

### 2.10 IL‐10 Regulates BMSCs’ Osteogenic‐Adipogenic Differentiation by Activating the PI3K‐Akt/Wnt Signaling Pathways

The extracellular matrix (ECM) is a core component of the stem cell niche that governs stem cell behavior and fate, modulating the transition from quiescence to a differentiation‐competent state and orchestrating lineage‐specific programs [38]. Our results confirmed a critical role for ECM‐receptor interaction in IL‐10‐regulated BMSC lineage specification. Because ECM‐receptor signaling is an established upstream environmental‐sensing module that integrates extracellular cues, we focused on the downstream PI3K/Akt and Wnt/β‐catenin pathways coupled to it. Western blot showed that IL‐10 significantly increased PI3K and Akt phosphorylation and β‐catenin (a key Wnt effector) expression under all three conditions (CM, OSTE, and ADI), indicating stable activation of the PI3K‐Akt and Wnt pathways across differentiation microenvironments (Figure 10A–F). To test their functional requirement, we blocked the PI3K‐Akt and Wnt pathways with specific inhibitors. Under osteogenic induction, the PI3K inhibitor LY294002 (10 µm), Akt inhibitor GSK690693 (5 µm), and β‐catenin inhibitor β‐catenin‐IN‐2 (10 µm) abolished the IL‐10‐induced increase in ALP and mineralized‐nodule formation (Figure 10G–I). Under adipogenic induction, these inhibitors reversed IL‐10's suppression of lipid‐droplet formation (Figure 10J,K). Western blot confirmed that inhibiting either pathway attenuated IL‐10‐induced osteogenic markers (ALP, RUNX2) and restored adipogenic markers (PPARγ, ADIPOQ) (Figure 10L–O).

**FIGURE 10:**
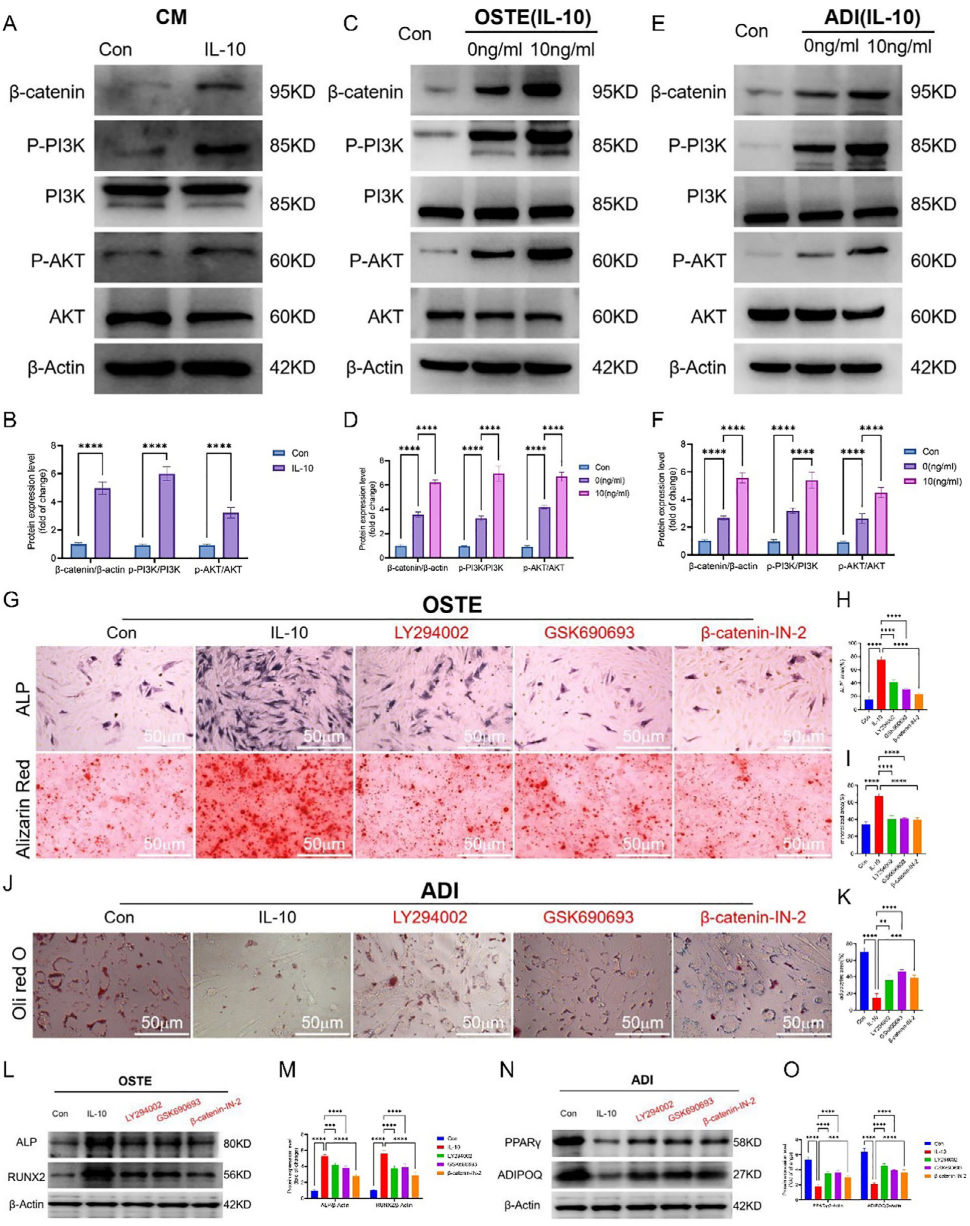
PI3K‐Akt/Wnt signaling is essential for IL‐10–mediated BMSC fate regulation. (A,C,E) Western blot analysis of PI3K‐Akt and Wnt signaling pathway molecules under CM (A), OSTE (C), and ADI (E) conditions. (B,D,F) Quantitative analysis of protein expression levels under CM (B), OSTE (D), and ADI (F) conditions. (G,J) Representative staining images of ALP/Alizarin Red (G, osteogenesis) and Oil Red O (J, adipogenesis) after treatment with pathway inhibitors. (H,I,K) Quantitative analysis of ALP area (H), mineralized area (I), and adipocyte area (K). (L,N) Western blot analysis of osteogenic (L) and adipogenic (N) marker genes after inhibitor treatment. (M,O) Quantitative analysis of osteogenic (M) and adipogenic (O) protein expression levels. For panel B, D, F, H, I, K, M, O: ^**^ *p*<0.01, ^****^ *p*<0.0001 by one‐way ANOVA analysis or two‐way ANOVA analysis. Data are presented as mean ± SD, *n* = 3/group.

## 3 Discussion

The interplay between immune modulation and stem cell fate is a central paradigm in regenerative medicine [39]. Here, IL‐10 acts through a dual axis, both suppressing pro‐inflammatory macrophage polarization and directly programming BMSC osteogenic commitment. These findings challenge the view of IL‐10 as merely an anti‐inflammatory cytokine, positioning it as a molecular switch that synchronizes immune homeostasis with metabolic reprogramming during xenogeneic scaffold integration.

The temporal dynamics of inflammation after biomaterial implantation critically determine regenerative outcomes [40, 41, 42]. Implantation inevitably elicits inflammation that can damage regenerating tissue and compromise transplanted cell viability [43], with macrophages as key mediators [44]. Our serological profiling reveals a biphasic cytokine cascade—an early pro‐inflammatory surge peaking at 1–2 weeks followed by gradual predominance of anti‐inflammatory mediators—consistent with the established concept of “inflammation‐resolution coupling” in tissue repair [45, 46, 47]. The distinctive contribution of our IL‐10‐loaded system is to compress the inflammatory window—not by eliminating the initial pro‐inflammatory phase, essential for pathogen clearance and recruitment of repair‐competent cells, but by accelerating resolution. The reduction in CD80‐positive macrophages in the IL‐10 group invites a nuanced interpretation: CD80, traditionally a canonical M1 marker, is increasingly seen as a dynamic co‐stimulatory molecule reflecting activation status rather than terminal differentiation [48]. Despite the limited availability of canine‐specific antibodies, our data suggest IL‐10 does not merely suppress macrophage activation but actively attenuates pro‐inflammatory signaling. Releasing IL‐10 from MPDA nanoparticles avoids excessive immunosuppression—which could compromise host defense—while ensuring adequate cytokine availability during peak inflammatory stress, an advance over static IL‐10 supplementation, which has shown limited clinical efficacy owing to narrow therapeutic windows and systemic side effects. The hierarchical sustained‐release platform combining MPDA with PNIPAM thermosensitive hydrogel is a core innovation of this work. Synthetic polymers with tunable degradation and customizable physicochemical properties are versatile carriers for sustained protein and cytokine delivery [49, 50]. Our MPDA‐in‐hydrogel carrier physically encapsulates IL‐10 and preserves its native conformation during matrix degradation, mitigating burst release and sustaining bioactivity, which represents a mature protein‐delivery strategy in synthetic polymer systems. Serum cytokine monitoring shows the BMSCs + IL‐10 group sustains a low pro‐inflammatory, high anti‐inflammatory phenotype. This reflects the unique “sense‐and‐respond” plasticity of BMSCs: quiescent BMSCs maintain only basal immunomodulatory activity but, once licensed by local pro‐inflammatory factors, rapidly upregulate effectors including IL‐10 to initiate spatiotemporally specific immunosuppression [51]. This lets BMSCs match the inflammatory timeline of bone regeneration: early on, nanoparticle‐released exogenous IL‐10 rapidly suppresses excessive inflammation, and later, as it is depleted, BMSCs continuously secrete endogenous IL‐10 and TGF‐β, establishing a seamless “exogenous initiation–endogenous maintenance” transition. This overcomes the short half‐life of recombinant IL‐10 and circumvents the delayed repair from the 7–14‐day inflammatory peak of xenogeneic bone, achieving a shift from conventional “structural substitution” to “functional immunomicroenvironment modulation.”

Perhaps the most provocative finding is that IL‐10 exerts direct, cell‐autonomous effects on BMSC differentiation, independent of its immunomodulatory functions. Transcriptomic analysis across basal, osteogenic, and adipogenic conditions reveals IL‐10 as a master regulator of the osteogenic‐adipogenic equilibrium, promoting Runx2‐driven osteogenesis and suppressing PPARγ‐mediated adipogenesis. This challenges the view of IL‐10 as solely an immune‐cell cytokine, revealing that it interfaces directly with mesenchymal stem cell signaling. The temporal specificity of IL‐10 during the early differentiation window is notable: by acting at lineage commitment rather than terminal differentiation, IL‐10 effectively “locks” BMSCs into an osteogenic trajectory, preventing the adipogenic drift that often compromises bone regeneration in pathological conditions. KEGG enrichment identifies ECM‐receptor interaction as a pivotal upstream sensing module that orchestrates downstream PI3K‐Akt and Wnt signaling to govern IL‐10‐mediated lineage commitment. As an essential niche component, the ECM regulates cellular behavior and fate [52], anchoring BMSCs and modulating proliferation, polarization, migration, and differentiation [38]. For example, hMSCs on 62–68 kPa ECM undergo osteogenic differentiation dependent on integrin α5, Akt, and GSK‐3β [53]. Notably, the PI3K‐Akt and Wnt pathways converge at GSK‐3β, which is inhibited by Akt‐mediated phosphorylation and is also a key component of the β‐catenin degradation complex [54]. Thus, IL‐10‐induced PI3K/Akt activation stabilizes β‐catenin by inhibiting its GSK‐3β‐dependent degradation, amplifying Wnt output. Functionally, blockade of PI3K (LY294002), Akt (GSK690693), or β‐catenin (β‐catenin‐IN‐2) abrogates IL‐10‐promoted osteogenesis and derepresses adipogenesis, showing that the ECM‐PI3K/Akt‐Wnt axis is a non‐redundant network for IL‐10‐mediated lineage commitment.

Several questions warrant future study. First, the precise link between IL‐10 receptor engagement and PI3K‐Akt/Wnt activation in BMSCs remains unclear. Although our Western blot data support pathway activation, the intermediary adaptors—such as IL‐10 receptor or PI3K regulatory subunits—and their cross‐talk with parallel cascades require systematic genetic and pharmacological dissection. We plan co‐immunoprecipitation (Co‐IP) with mass spectrometry to screen adaptor proteins interacting with the IL‐10 receptor and small interfering RNA (siRNA) knockdown to verify the function of core receptor subunits in the PI3K‐Akt/Wnt cascades. Second, the long‐term in vivo fate of the MPDA and PNIPAM components—biodegradation kinetics, tissue distribution, and potential systemic accumulation—requires thorough toxicological evaluation. We will use long‐term fluorescent tracing to monitor MPDA and PNIPAM degradation and distribution over 12 months post‐implantation, with blood counts, serum biochemistry, and major‐organ histopathology to assess systemic biosafety. Third, scaling from preclinical canine models to human use will require optimizing IL‐10 loading, release kinetics, and scaffold parameters (porosity, interconnectivity, mechanical properties) to match human craniofacial defect dimensions and healing dynamics. Conventional static 3D‐printed scaffolds cannot adapt to progressive shifts in the post‑implantation bone microenvironment. Four‐dimensional (4D) bioprinting, which adds time as a fourth dimension to 3D bioprinting, has emerged as a next‐generation tissue‐engineering approach capable of building complex, functional structures [55, 56, 57], giving scaffolds biomimetic adaptability to tissue remodeling. For clinical translation, we propose two improvements: tuning IL‑10 loading and release to human inflammatory patterns and fabricating multilayered, time‑responsive composite scaffolds by 4D bioprinting. Such next‑generation skull biomaterials would enable precise spatial patterning and staged immunomodulation, advancing this immuno‑metabolic strategy.

In conclusion, we establish a proof of concept for “immuno‐metabolic tissue engineering,” in which a single factor, IL‐10, both remodels the inflammatory milieu and directs stem cell fate. By uniting temporal immune control with direct BMSC fate programming, these findings advance IL‐10 biology and offer a translational roadmap for bone‐regeneration strategies centered on immune–stem cell cross‐talk.

## 4 Experimental Section

### 4.1 Experimental Animals

All animal care, experimental and surgical processes and postoperative euthanasia comply with the ARRIVE guidelines and were performed in strict accordance with the ethical principles of the NIH Guide for the Care and Use of Laboratory Animals (NIH Publications No. 8023, revised 1978). All animal procedures were performed in accordance with the Guide for the Care and Use of Laboratory Animals and approved by the Animal Care and Use Committee of Air Force Medical University (IACUC‐20210120). Male beagle dogs (18–20 months old, 8–12 kg) were provided by Dilepu Biomedical Co., Ltd. BALB/c Nude mice were purchased from GemPharmatech (Chengdu) Co., Ltd. Animals were housed at 24°C–26°C, 40%–70% humidity, and a 12 h light/dark cycle with free access to food and water.

### 4.2 Experimental Groups

In vivo skull defect repair study: Adult beagle dogs were randomly assigned to five groups (*n* = 3 per group): (1) Blank group: skull defect without cranioplasty; (2) Xenograft group: cranioplasty using porous xenogeneic porcine skull scaffolds alone; (3) IL‐10 group: cranioplasty with xenogeneic scaffolds loaded with PNIPAM thermosensitive hydrogels containing MPDA encapsulated IL‐10; (4) BMSCs group: cranioplasty with xenogeneic scaffolds loaded with PNIPAM thermosensitive hydrogels carrying canine BMSCs; (5) BMSCs + IL‐10 group: cranioplasty with xenogeneic scaffolds loaded with PNIPAM thermosensitive hydrogels containing MPDA‐encapsulated IL‐10 and canine BMSCs.

Nude mouse subcutaneous transplantation study: nude mice were randomly divided into two subcutaneous transplantation groups (*n* = 3 per group): (1) BMSCs group: subcutaneous implantation of PNIPAM thermosensitive hydrogels loaded with canine BMSCs alone; (2) BMSCs + IL‐10 group: subcutaneous implantation of PNIPAM thermosensitive hydrogels loaded with MPDA@IL‐10 and canine BMSCs.

In vitro mechanistic investigation: For RNA‐sequencing (RNA‐seq), canine BMSCs were randomly divided into six subgroups based on two variables (culture medium type and IL‐10 treatment): (1) CM group: basal culture medium with or without IL‐10; (2) OSTE group: osteogenic induction medium with or without IL‐10; (3) ADI group: adipogenic induction medium with or without IL‐10.

### 4.3 Establishment of Skull Defect Model and Graft Implantation

To establish canine skull defect models and perform cranioplasty, the team was divided into two groups: one preparing the bioactive repair materials and the other performing the critical‐sized skull defect surgery on beagle dogs. Xenogeneic scaffolds were freshly fabricated in the operating room under aseptic, temperature‐controlled conditions and immediately implanted to preserve bioactivity. Beagles fasted 12 h with 2 h water restriction preoperatively. IM dexmedetomidine (5–10 µg/kg) plus methadone/morphine (0.3 mg/kg) provided sedation and preemptive analgesia. Anesthesia was induced with IV propofol (2–4 mg/kg), maintained with isoflurane in oxygen via circle breathing system to preserve cerebral autoregulation. Vital signs (heart rate, respiratory rate, rectal temperature, and pulse oxygen saturation) were continuously monitored to assess anesthetic depth and maintain stable circulation, respiration, and temperature. Once a stable anesthetic plane with adequate muscle relaxation and loss of pain reflex was achieved, dogs were positioned prone with the head immobilized, and the surgical area was aseptically prepared, disinfected, and draped to expose the skull. A 2.0‐cm‐diameter full‐thickness critical‐sized defect was created at a predefined site on the right parietal bone with a sterile dental drill under continuous saline irrigation to prevent thermal injury while preserving the dura mater. Per grouping, cranioplasty materials were implanted and sutured in place, and the periosteum was continuously sutured for added fixation. The incision was closed layer by layer and disinfected. Postoperatively, daily wound care and disinfection were performed, and subcutaneous broad‐spectrum antibiotics were given for 7 days to prevent infection. General condition, food intake, activity, and wound healing were monitored to ensure recovery without redness, exudation, or infection. All animals received postoperative pain management: meloxicam (0.2 mg/kg SC) 30 min before surgery completion, then every 24 h for 5 days, with buprenorphine (0.01 mg/kg SC) every 12 h for the first 72 h. Veterinarians performed twice‐daily standardized pain scoring for 7 days, with rescue buprenorphine for moderate or severe pain. All animals were euthanized 6 months after surgery: sedation was induced with intramuscular medetomidine (10 µg/kg) and morphine (0.3 mg/kg), followed by intravenous propofol (2 mg/kg) for deep anesthesia, with the absence of corneal and pain‐withdrawal reflexes confirmed. An intravenous overdose of sodium pentobarbital (200 mg/kg) was then administered [58]; skull specimens with grafts were harvested, rinsed with saline, and fixed in 4% paraformaldehyde for later analysis.

### 4.4 BMSCs Preparation

Under stable anesthesia, dogs were placed prone; the thigh was shaved and disinfected with povidone‑iodine. The skin was incised and subcutaneous tissue and muscle bluntly dissected to expose the femur. A hole was drilled through the femoral cortex, and ∼12 mL of bone marrow was slowly aspirated with a heparinized syringe. The hole was sealed with bone wax, and the tissues were closed layer by layer. The marrow was divided into three 4‑mL aliquots and mixed with an equal volume of complete medium (BMDG‐G101, HyCyte) pre‑warmed to 37°C. Diluted marrow was then overlaid onto 4 mL canine mononuclear cell separation medium (LDS1077, TBD) in a 15‑mL tube. After centrifugation at 2000 rpm for 20 min, the grayish‑white mononuclear cell layer was collected, resuspended in complete medium, and seeded in 10‑cm dishes at 37°C with 5% CO_2_. Non‐adherent cells were removed at medium changes, and adherent BMSCs were subcultured 1:3. Only passage 3 BMSCs were used for all in vitro and in vivo experiments to ensure a uniform phenotype across groups.

### 4.5 In Vitro Cell Differentiation

Osteogenic induction: When BMSCs reached 70%–80% confluence, the complete medium (BMDG‐D101, HyCyte) was replaced with osteogenic induction medium. The induction medium was refreshed every 2–3 days, and the induction period lasted for 21–28 days. During the induction process, the morphological changes of cells were observed regularly under an inverted microscope. After the induction was completed, ARS was performed to identify calcium nodule formation, which is a specific marker of osteogenic differentiation; positive cells or nodules would be stained red.

Adipogenic induction: BMSCs were cultured to 70%–80% confluence and replaced with adipogenic differentiation medium maintenance medium (BMDG‐D102, HyCyte). After 1 day of culture in maintenance medium, BMSCs were replaced with adipogenic differentiation induction medium and continued for 3 days. They were induced for 14–21 days according to the above replacement frequency, and cell morphological changes were observed. During induction, the formation of lipid droplets in cells was observed periodically. After induction, oil red O staining was carried out: the cells were fixed, stained with oil red O working solution, and then decolorized appropriately; the lipid droplets in adipocytes would be stained bright red, indicating successful adipogenic differentiation.

Chondrogenic induction: BMSCs were cultured until they reached 70%–80% confluence, and then the complete medium was replaced with chondrogenic induction medium (BMDG‐D203R, HyCyte). The induction medium was refreshed every 2–3 days, and the induction period was 21–28 days. After induction, alcian blue staining was performed to detect the synthesis of glycosaminoglycans (a key component of cartilage matrix); positive chondrogenic cells and matrix would be stained blue, confirming successful chondrogenic differentiation.

### 4.6 ALP Staining

At the predetermined induction time point, the medium was aspirated, and cells were rinsed once with 1×phosphate‐buffered saline (PBS). Cells were then fixed with 4% neutral formaldehyde at room temperature for 30–60 min. The fixative was removed, and the cells were washed twice with 1×PBS. For ALP staining, the procedure followed the NBT/BCIP kit instructions (C3206, Beyotime). Images were acquired using the EVOS M5000 imaging system (Thermo Fisher Scientific, USA) and analyzed in ImageJ software (v1.8.0; NIH, Bethesda, MD, USA).

### 4.7 Transwell Invasion Assays

For invasion assays, 8.0 µm Transwell inserts (3422, Corning) were precoated with 1:8 diluted Matrigel (C0371‐1 mL, Beyotime) and polymerized at 37°C for 2–3 h. Serum‐starved cells (5 × 10^5^ cells/mL in 200 µL serum‐free medium) were seeded in the upper chamber, with 600 µL complete medium (10% FBS) in the lower chamber. After 24–48 h, invaded cells were fixed with 4% paraformaldehyde (G1101, Servicebio), stained with 0.1% crystal violet (G1063, Solarbio), and counted in five random fields.

### 4.8 In Vitro Biocompatibility Assessment

PNIPAM Hydrogel Extract: Following ISO 10993‐12:2021 (Clause 7.2.2), extraction was performed at 0.1 g/mL mass/volume ratio for porous materials using canine BMSC‐specific medium for 72 h (Clause 7.3.2) at 37°C, 5% CO_2_, 60 rpm. Extracts were centrifuged (3000 rpm, 10 min) and filtered (0.22 µm). Bone‐Scaffold Extract and PNIPAM@MPDA@IL‐10 Complex Extracts: Similarly prepared per ISO 10993‐12:2021 at 0.1 g/mL for 72 h, centrifuged (3000 rpm, 15 min), and filtered. Biocompatibility test: Following ISO 10993–5:2009 (Clauses 8.1–8.3), canine BMSCs were seeded at 1 × 10^4^ cells/well. After 24 h of attachment, the medium was replaced with 100% extract or blank control (fresh medium).

### 4.9 Flow Cytometry

Regarding cell‐cycle detection, BMSCs in the logarithmic growth phase were harvested, washed twice with PBS, and fixed overnight at 4°C in 70% ethanol. After fixation, cells were washed twice with PBS, treated with RNase A (EN0531, Thermo Fisher Scientific) at 37°C for 30 min, and stained with propidium iodide (PI, P4170, Sigma–Aldrich) on ice for 30 min in the dark.

For flow cytometry identification of BMSCs, cells were harvested, washed with PBS, and resuspended at 1 × 10^6^ cells/mL. Aliquots of 1 × 10^6^ cells were incubated with fluorescently labeled antibodies—CD90‐paired‐end (PE) (12‐5900‐42, Thermo Fisher Scientific), CD44‐FITC (11‐5440‐42, Thermo Fisher Scientific), CD34‐ PE (MA1‐81855, Thermo Fisher Scientific), and CD14‐ FITC (MA1‐82074, Thermo Fisher Scientific)—at 4°C in the dark for 30 min. After PBS washing, cells were resuspended in PBS and analyzed by flow cytometry. The gating strategy was performed as follows: Cell populations of interest were first distinguished on the FSC‐A vs. SSC‐A dot plot; singlet gating (FSC‐H vs. FSC‐A) was then implemented to eliminate cell doublets and aggregates for single‐cell analysis. Isotype control samples were utilized to define the threshold of background fluorescence (restricted within approximately 1%–2% positive events). Under the threshold criteria calibrated by isotype controls, the positive expression rates of BMSC surface markers (CD90, CD44, CD34, CD14) were quantified separately.

For peripheral blood T cell subset analysis, heparin‐anticoagulated whole blood was collected from beagle dogs at predetermined postoperative time points. Two canine‐specific fluorochrome‐conjugated antibodies were utilized for immunostaining, including anti‐CD4‐ PE‐Cyanine7 (25‐5040‐42, Thermo Fisher Scientific) and anti‐CD8‐APC (17‐5080‐42, Thermo Fisher Scientific). Briefly, 5 µL of each antibody was mixed with 100 µL heparinized whole blood and incubated at room temperature for 30 min; then 500 µL erythrocyte lysis buffer was added for 15 min at room temperature. Cells were washed twice with 2 mL PBS, and pellets were resuspended in 500 µL PBS before flow cytometry. The gating strategy was carried out as follows: leukocyte populations were initially identified on the FSC‐A vs. SSC‐A dot plot to isolate lymphocytes; FSC‐H vs. FSC‐A singlet gating was applied to eliminate cell doublets and aggregates; and a separate 7‐AAD staining tube was prepared to retain only 7‐AAD^−^ viable cells for subsequent analysis. An unstained blank control tube was prepared to define the threshold cross gate of negative populations, while CD4‐PE‐Cyanine7 single‐stained tube and CD8‐APC single‐stained tube were respectively set to calibrate fluorescence compensation between PE‐Cyanine7 and APC channels. Acquisition began only after baseline gates and spectral compensation were calibrated with control tubes.

### 4.10 EDU Assays

Cell proliferation was assessed using the BeyoClick EdU‐488 Cell Proliferation Kit (C0071S, Beyotime) according to the manufacturer's instructions. Briefly, cells were seeded at appropriate density in 6‐well plates and allowed to attach overnight. Following drug treatment, cells were incubated with EdU working solution for 4 h to label proliferating cells. After EdU labeling, culture medium was aspirated and cells were fixed with 4% paraformaldehyde at room temperature for 15 min. Fixed cells were washed three times with PBS containing 3% BSA for 3–5 min each, followed by permeabilization with PBS containing 0.3% Triton X‐100 at room temperature for 10–15 min. Cells were then washed 1–2 times with wash buffer for 3–5 min each, and subsequently incubated with DAPI solution at room temperature for 10 min protected from light. After three washes, cells were visualized under a fluorescence microscope. The percentage of EdU‐positive cells was calculated as follows: (EdU‐positive cells/DAPI‐positive total cells) × 100%.

### 4.11 Live/dead Cell Staining

Live/dead cell staining was performed using the Calcein‐AM/PI Double Staining Kit (C2015M, Beyotime). Cells were seeded at appropriate density in 24‐well plates and allowed to attach or subjected to treatment. After removing the culture medium and washing twice with PBS, cells were incubated with Calcein‐AM/PI working solution at 37°C for 20–30 min protected from light. Unbound dyes were removed by washing twice with PBS. Fluorescent images were acquired using an inverted fluorescence microscope. Live cells exhibited green fluorescence, while dead cells showed red fluorescence. Cell viability (%) was calculated as (live cells/total cells) × 100%.

### 4.12 In Vitro Hydrogel Degradation Assay

Equal‐volume cylindrical hydrogel constructs were fully gelled at 37°C and immersed in sterile PBS (pH 7.4) for in vitro hydrolytic degradation. Samples were incubated at 37°C throughout. At predetermined intervals, specimens were retrieved from the medium. Excess surface salts were rinsed off with deionized water, and surface water was blotted with filter paper before weighing. The residual mass retention rate (%) of hydrogels was calculated using the following formula: Mass retention rate (%) = W _t_ / W _0_ ×100, where W _t_ ​represents the residual weight of the hydrogel at each designated degradation time point and W _0_ denotes the initial weight of the intact hydrogel before degradation. All weights were measured on a high‐precision analytical balance (BSA124S‐CW), with three biological replicates per group.

### 4.13 Bioactivity Assessment of Released IL‐10

To verify that IL‐10 released from the PNIPAM@MPDA@IL‐10 composite retained its immunomodulatory bioactivity, an in vitro macrophage polarization assay was performed with the murine macrophage line RAW 264.7. Cells were maintained in DMEM with 10% FBS and 1% penicillin‐streptomycin at 37°C with 5% CO_2_. For M1 polarization, RAW 264.7 cells were seeded in 6‐well plates at 2 × 10^5^ cells/well and stimulated with lipopolysaccharide (LPS, 100 ng/mL) for 24 h. After M1 polarization, cells were randomly divided into three groups: ① M0 control: untreated RAW 264.7 cells. ②M1 control: medium alone; ③M1 + released IL‐10: IL‐10 released from the PNIPAM@MPDA@IL‐10 composite at day 7 (diluted to 10 ng/mL equivalent based on BCA quantification). After 24 h of treatment, cells were harvested for flow cytometric analysis, and pro‐inflammatory cytokines TNF‐α (ab108910; Abcam) and IL‐1β (ab197742; Abcam) in the supernatant were detected using ELISA.

### 4.14 Fabrication of Nanocomposite Hydrogel

IL‐10‐loaded MPDA nanoparticles were prepared as follows. Briefly, 30 mg MPDA (RuixiBiotech Co., Ltd.) was dispersed in 10 mL deionized water, and 1 mg recombinant IL‐10 (Cat: 62000‐WNAE, Sino Biological) was added. The mixture was stirred at room temperature for 24 h to let IL‐10 adsorb into the mesopores. Unloaded IL‐10 was removed by centrifugation, and the resulting MPDA@IL‐10 nanoparticles were washed three times with deionized water. For the preparation of thermosensitive hydrogel composites, 3 g of PNIPAM (RuixiBiotech Co., Ltd.) was dissolved in 30 mL of deionized water to form a 10% (w/v) PNIPAM hydrogel solution. Subsequently, the entire batch of MPDA‐IL‐10 nanoparticles (equivalent to 30 mg of initial MPDA) was added into the PNIPAM solution under gentle magnetic stirring, yielding a homogeneous MPDA‐IL‐10/PNIPAM nanocomposite hydrogel. The size and zeta potential of MPDA@IL‐10 nanoparticles were measured using a nanoparticle size and zeta potential analyzer (NanoBrook 90plus PALS, Brookhaven, USA). Ultrasonic cleaning was performed with an ultrasonic cleaner (JP‐020, Jiemeng, Shenzhen, China). The concentration of IL‐10 was quantified using a multifunctional microplate reader (Infinite E Plex, Tecan, Switzerland). The morphological and structural features of the nanoparticles and hydrogel composites were characterized by SEM (Sigma 300, ZEISS, Germany) and TEM (TF20, FEI, USA). The rheological properties of the PNIPAM‐based hydrogels were evaluated using a rheometer (MARS60, Haake, Germany). The supernatant containing unbound free IL‐10 was collected for total protein quantification using a commercial BCA protein assay kit. A serial dilution of standard solution was prepared to construct a linear standard curve, and the total mass of free IL‐10 in supernatant was calculated by absorbance fitting. Free IL‐10 mass W _free drug_ was calculated from the standard curve. Where W _total_​ = 1 mg (initial IL‐10 input), W _MPDA_​ = 30 mg. Loaded drug mass: W loaded drug ​ = W _total_ −W _free drug_. Drug Loading Content (DLC, %): W _loaded drug_ / (W _nanoparticles_ +W _loaded drug_) ×100%. Encapsulation efficiency (EE, %) = (W _loaded drug_ /W _total_) ×100%. The IL‐10 concentration *C_t_* ​ measured at each time point directly reflects the amount of free IL‐10 released into the medium at that moment. The release percentage was calculated as R _t_​ = (C_t_ × V) ​/ W _loaded_ ×100%. Where C_t_​ is the IL‐10 concentration at time t determined by BCA (mg/mL), V is the release medium volume, and W _loaded_ is the total IL‐10 mass in the loaded system. The release conditions are PBS (pH 7.4) and 37 °C constant temperature oscillation (60 rpm). The final drug loading of the hydrogel containing MPDA@IL‐10 was 641 ug/30 mL.

### 4.15 In Vivo Transplantation

According to the experimental design, nude mice were subcutaneously injected with hydrogel‐loaded BMSCs and hydrogel‐loaded MPDA@IL‐10 + BMSCs. At the scheduled time, the skin and subcutaneous tissue were separated, the tissue block was removed and fixed in 4% paraformaldehyde at 4°C for 48 h, and H&E and immunofluorescence staining were performed.

### 4.16 SEM Analysis

Samples were fixed with electron microscopy fixative (G1102, Servicebio) at room temperature for 2 h, washed three times with 0.1 m PB (pH 7.4) for 15 min each, post‐fixed with 1% OsO_4_ (18456, Ted Pella Inc.) at room temperature for 1–2 h protected from light, and washed three times with PB. Samples were dehydrated through a graded ethanol series (30%, 50%, 70%, 80%, 90%, 95%, and 100%) for 15 min each, followed by isoamyl acetate transition for 15 min and critical point drying (Quorum, K850). Dried specimens were mounted on stubs using carbon adhesive tabs, sputter‐coated with gold for 30 s (HITACHI, MC1000), and examined under a scanning electron microscope (HITACHI, SU8100).

### 4.17 Preparation of Xenogeneic Bama Mini‐Pig Skull Scaffolds

Bama mini‐pig skulls were harvested under sterile conditions, and all adherent soft tissue (periosteum and connective tissue) was excised with sterile scissors and forceps without damaging the bone matrix. Cleaned skulls were trimmed into 2.0‐cm‐diameter circular slices with a precision cutter. Before delipidation and deproteinization, uniform single cortical perforations were drilled in each slice to create a homogeneous porous structure for cell accommodation, vascular ingrowth, and nutrient transport. Slices then underwent standardized delipidation and deproteinization: immersion in methanol/chloroform (1:1, v/v) at room temperature for 8 h to remove lipids and lipophilic impurities. The solvent was discarded, and slices were ultrasonically cleaned in deionized water at 50 °C for 40 min to remove residual solvent and debris. Slices were then soaked in 3% (v/v) hydrogen peroxide (H_2_O_2_) at room temperature for 48 h to remove soluble proteins, antigens, and cellular fragments, reducing immunogenicity. Slices were rinsed with deionized water until neutral and air‐dried on a sterile, clean bench, preserving the porous matrix. Scaffolds were terminally sterilized by ^6^ ^0^Co gamma irradiation at 25 kGy and stored in sterile sealed containers. The workflow was repeated for three independent batches. Batch‐to‐batch variation in residual dsDNA, α‐Gal, and SLA antigen levels was minimal (Figure S2), with small inter‐batch standard deviations confirming excellent process stability and reproducibility.

### 4.18 Residual DNA Assay

Quantification of residual DNA in xenogeneic bone before and after delipidation and deproteinization was performed with the PicoGreen dsDNA Reagent. Equal weights of untreated and processed bone were homogenized for total DNA extraction using standard lysis protocols. Serially diluted dsDNA standards were mixed with PicoGreen working solution to generate a standard calibration curve. Extracts were mixed with the reagent and incubated in the dark, and fluorescence was measured on a microplate reader to calculate residual DNA per gram of bone.

### 4.19 3D Printing

3D bioprinting achieved uniform cell distribution within the scaffold. Scanned data were reconstructed in Cura 5.01 (Ultimaker, Netherlands), and pore coordinates were marked. The G‐code was manually edited to restrict bioink deposition to specific coordinates by disabling extrusion during nozzle travel. A multi‐nozzle bioprinter (LivPrint, Elite, Medprin, China) with a screw‐driven extruder and temperature‐controlled printhead and stage was used for deposition. Bioink was prepared by resuspending BMSCs (6 × 10^6^ cells/mL) and MPDA@IL‐10 nanoparticles (21.3 µg/mL) in 10% (w/v) PNIPAM precursor at 4°C. The printing parameters were optimized as follows: nozzle temperature, 15°C; platform temperature, 37°C; nozzle inner diameter, 25 G (inner diameter ∼260 µm, thin‐wall); screw linear extrusion speed, 0.05 mm/s (equivalent to 0.05 mL/min or 0.9 µL/s, delivering 1 mL bioink per 18 min); idle travel speed (non‐printing), 6 mm/s Following the programmed trajectory, 20 µL of BMSCs/MPDA@IL‐10 bioink was deposited per pore. Each scaffold comprised approximately 17 pores, yielding a total cellular load of 2 × 10^6^ BMSCs and 0.15 µg IL‐10 per scaffold (calculated as 6 × 10^6^ cells/mL × 0.02 mL/pore × 17 pores = 2.04 × 10^6^ cells and 21.3 µg/mL × 2.09% × 0.02 mL/pore × 17 pores = 0.15 µg IL‐10). Scaffolds were incubated at 37°C with 5% CO_2_ for 24 h for gelation and cell attachment before implantation.

### 4.20 CT Testing

For dynamic monitoring of skull bone repair in beagle dogs, multi‐detector spiral CT scanning was performed monthly using a clinical CT scanner (Model 760, United Imaging, China). The dogs were anesthetized with the same combined anesthesia protocol as the surgical procedure to ensure immobility during scanning, then placed in a prone position with the head fixed to avoid displacement and ensure the consistency of scanning positions at each time point. The scanning region was precisely positioned at the 2.0 cm critical‐sized skull defect site, and standardized CT scanning parameters were adopted for axial continuous scanning of the skull to obtain clear tomographic images of the defect repair area.

### 4.21 ELISA

Serum cytokine levels in beagle dogs from each experimental group at different postoperative time points were detected by ELISA strictly following the instructions of the corresponding ELISA kits: peripheral blood was collected from experimental dogs at each postoperative time point, placed in anticoagulant‐free collection tubes, allowed to clot naturally at room temperature, and then centrifuged at 4°C and 3000 rpm for 15 min to separate the serum. The serum was aliquoted and stored at −80°C; before detection, it was thawed at room temperature and re‐processed by centrifugation. All reagents and coated strips of the kit were balanced at room temperature for 30 min, and gradient standards were added to the enzyme‐labeled plate. The absorbance (OD value) of each well was measured at the specified wavelength using a microplate reader. A standard curve was plotted with the standard concentration as the abscissa and the corresponding OD value as the ordinate. The concentrations of TNF‐α, IL‐8, IL‐10, IL‐6, and TGFβ in each serum sample were calculated according to the standard curve. The experiment was repeated three times, and the average value was taken as the final result. The following commercial ELISA kits were used in this study: Canine TNF‐alpha Quantikine ELISA Kit (CATA00; R&D Systems), Canine IL‐10 Quantikine ELISA Kit (CA1000; R&D Systems), Canine IL‐6 Quantikine Kit (CA6000; NOVUS), Canine TGF‐beta Quantikine ELISA Kit (PDB100C; NOVUS), Canine IL‐8 Quantikine ELISA Kit (CA8000; NOVUS).

### 4.22 Micro‐CT Testing

For micro‐CT testing, fixed skull specimens from beagle dogs were scanned (voltage, 80 kV; current, 0.06 mA; integration time, 500 ms; voxel size, 0.05 mm) on an NMC‐200 (PINGSENG Healthcare Inc., Kunshan, China), with the scanning region at the 2.0 cm critical‐sized defect. Raw data were collected with Cruiser software, reconstructed into three‐dimensional (3D) tomographic images with Recon software, and visualized, measured, and analyzed with Avatar software to obtain BMD and BV/TV of the defect area, with all tests repeated three times.

### 4.23 qPCR Analysis

Total RNA was extracted with TRIzol and reverse‐transcribed with a commercial kit. qPCR used SYBR Green Master Mix with gene‐specific primers on a real‐time PCR system. The thermal cycling conditions consisted of an initial denaturation at 95°C for 10 min, followed by 40 cycles of 95°C for 15 s and 60°C for 60 s. GAPDH served as the internal reference gene. Relative expression was calculated by the 2^−ΔΔCt method as fold change vs. control, with all samples in triplicate. The primers for mRNA were as follows: OCN‐F: “CTCAACCCCAACTGTGACGA”, OCN‐R: “AGCTGTGATGACAAGGACCC”; RUNX2‐F: “CGGAGTGGAAGAGGCAAGAG”, RUNX2‐R: “CGGGGTCCATCCACTGTAAC”; SP7‐F: “TGGAACAGAGTGGAGGAAGC”, SP7‐R: “AGTATGGCTTCTTTGCGCCT”; CEBPA‐F: “CGGGATCTCCAGGCTGC”, CEBPA‐R: “GGTATCCTTAATACTAGAGTTGCCA”; PPARγ‐F: “TGGGTGAAACTCTGGGAGAT”, PPARγ‐R: “TGTGTCAACCATGGTAGTTTCTTG”; Fabp4‐F: “TGAAAGAAGTGGGAGTGGGC”, Fabp4‐R: “CCTGGCCCAGTTTGAAGGAA”; and GAPDH‐F: “TCCATCTTCCAGGAGCGAGA”, GAPDH‐R: “GGTTCACGCCCATCACAAAC”.

### 4.24 Western Blot

BMSCs were lysed on ice in RIPA buffer with protease and phosphatase inhibitors; protein was quantified by BCA assay (AccuRef Scientific, Xi'an, China), separated by sodium dodecyl sulfate‐polyacrylamide gel electrophoresis (SDS‐PAGE), and transferred to polyvinylidene fluoride (PVDF) membranes. Membranes were blocked with 5% non‐fat milk for 1 h, incubated with primary antibodies overnight at 4 °C, washed three times in TBST, and incubated with horseradish peroxidase (HRP)‐conjugated secondary antibodies for 1 h at room temperature. Bands were visualized with an enhanced chemiluminescence (ECL) kit and quantified in ImageJ with β‐actin as the reference. The following antibodies were used: rabbit anti‐ALP (1:1000; NBP3‐25356; Novus), rabbit anti‐RUNX2 (1:1000; NBP2‐24755; Novus), rabbit anti‐PPARγ (1:1000; NB120‐19481; Novus), rabbit anti‐ADIPOQ (1:100; DF7000; Affinity), rabbit anti‐β‐catenin (1:1000; NBP1‐32239; Novus), rabbit anti‐PI3K (1:1000; AF6241; Affinity), rabbit anti‐Phospho‐PI3K (1:1000; AF3242; Affinity), rabbit anti‐Phospho‐AKT (1:1000; AF0016; Affinity), mouse anti‐AKT (1:1000; NBP2‐88960; Novus), and mouse anti‐β‐ACTIN (1:10,000; NB600‐501; Novus).

### 4.25 H&E Staining

Decalcified skull tissues were paraffin‐embedded and sectioned. Following deparaffinization and rehydration, sections were stained with hematoxylin (5–10 min), differentiated in 1% acid alcohol, blued, and counterstained with eosin (3–5 min). After dehydration and clearing, sections were mounted with neutral balsam.

### 4.26 Masson Staining

Decalcified skull tissues were paraffin‐embedded, sectioned, and stained with Masson's trichrome according to standard protocols. Briefly, sections were deparaffinized, rehydrated, and sequentially stained with Weigert's iron hematoxylin, ponceau fuchsin, and aniline blue with appropriate differentiation, then dehydrated, cleared, and mounted with neutral balsam.

### 4.27 Immunofluorescence and Immunohistochemistry

For immunofluorescence staining, skull tissues were decalcified, paraffin‐embedded, and sectioned; sections were deparaffinized in xylene and rehydrated through a graded ethanol series (100%, 95%, 85%, 75%) to distilled water, followed by heat‐mediated antigen retrieval in citrate buffer (pH 6.0). After natural cooling to room temperature, the sections were rinsed with 1×PBS, blocked with 5% bovine serum albumin for 1 h at room temperature to eliminate non‐specific binding, and incubated with the corresponding primary antibodies overnight at 4°C. The sections were then washed three times with 1×PBS, incubated with fluorescently labeled secondary antibodies for 1 h at room temperature in the dark, and counterstained with DAPI for nuclear labeling after another three washes with 1×PBS. Finally, the sections were sealed with anti‐fluorescence quenching mounting medium, and fluorescent images were collected and analyzed under an EVOS M5000 fluorescence microscope. The following antibodies were used: GS‐IB_4_ (1:100, I21411, Thermo Fisher Scientific), CD80 Monoclonal Antibody, FITC (1:100,11‐0801‐82, Thermo Fisher Scientific), rabbit anti‐CD206 (1:100; GTX03378, GeneTex), rabbit anti‐TNF‐α (1:100; NBP1‐19532SS; Novus), rabbit anti‐IL‐1β (1:100; NB600‐633; Novus), rabbit anti‐OPN (1:100; NBP3‐20504; Novus), rabbit anti‐RUNX2 (1:100; NBP2‐24755; Novus), rabbit anti‐Col1α1 (1:100; NBP1‐30054; Novus), mouse anti‐OCN (1:100; MA1‐20786; Thermo Fisher Scientific), rabbit anti‐Perilipin‐1 (1:100; ab3526; abcam), rabbit anti‐ADIPOQ (1:100; DF7000; Affinity), goat anti‐rabbit IgG (H + L), highly cross‐adsorbed secondary antibody, Alexa Fluor Plus 488 (1:1000; A11034; Invitrogen); and goat anti‐mouse IgG (H + L), highly cross‐adsorbed secondary antibody, Alexa Fluor Plus 555 (1:1000; A32727; Invitrogen).

For immunohistochemistry, paraffin sections underwent antigen retrieval, blocking, and overnight incubation with primary antibody at 4°C with mouse anti‐HLA class I ABC (1:1000, 66013‐1‐Ig, Proteintech) [59, 60] or anti‐HLA‐DR/DP (1:1000, MA1‐19145, Thermo Fisher Scientific). After PBS washing, sections were incubated with enzyme‐conjugated secondary antibodies for 30–60 min at room temperature, developed with DAB, counterstained with hematoxylin, dehydrated, cleared, and mounted. Stained sections were examined by light microscopy.

### 4.28 RNA‐seq and Transcriptome Analysis

Total RNA was extracted, purified, and used for library construction, followed by PE sequencing on the Illumina platform. Raw data were processed to obtain gene counts, and differentially expressed genes (DEGs) were identified with DESeq2. DEGs were defined as |log2FoldChange| ≥ 1 and P < 0.05, with the false discovery rate obtained by correcting the p‐value; fold change was log‐transformed (logFC), and genes with larger |logFC| and smaller P were more significantly differentially expressed. Volcano plots displayed DEG distribution by logFC and p‐value. DEGs were then analyzed by GO enrichment (biological process, cellular component, and molecular function) and Kyoto Encyclopedia of Genes and Genomes pathway enrichment. All raw RNA sequencing raw data generated in this study have been deposited in the National Center for Biotechnology Information (NCBI) Sequence Read Archive (SRA) public database under the following BioProject accession numbers: PRJNA1481046 (BMSCs under osteogenic induction), PRJNA1482497 (normal cultured BMSCs), PRJNA1482529 (BMSCs under adipogenic induction). The sequencing datasets are publicly accessible via the NCBI SRA platform without access restrictions upon manuscript online publication.

### 4.29 Signaling Pathway Inhibition Assays

To validate IL‐10's regulation of PI3K‐Akt/Wnt signaling during BMSC lineage commitment, selective small‐molecule inhibitors were applied using standardized pretreatment protocols [61, 62, 63]. Three inhibitors were utilized: the PI3K inhibitor LY294002 (final concentration, 10 µm, catalog HY‐10108, MedChemExpress), the Akt inhibitor GSK690693 (final concentration, 5 µM, catalog HY‐10249, MedChemExpress), and the β‐catenin inhibitor β‐catenin‐IN‐2 (final concentration, 10 µm, catalog HY‐136464, MedChemExpress). All inhibitors were dissolved in dimethyl sulfoxide (DMSO) to prepare 10 mm stock solutions, which were stored at −20°C and protected from light. For rescue experiments, BMSCs were pre‐incubated with the inhibitor (in complete medium; DMSO ≤0.1%) for 2 h before IL‐10 (10 ng/mL) stimulation and culture in induction medium, with fresh inhibitor added every 48 h over the 7‐day differentiation.

### 4.30 Statistical Analysis

Data are presented as mean ± standard deviation (SD) and analyzed in GraphPad Prism. Two groups were compared by unpaired Student's *t*‐test, multiple groups by one‐way analysis of variance (ANOVA) with Tukey's post‐hoc test, and two‐variable analyses by two‐way ANOVA. P < 0.05 was considered significant, and all experiments were independently repeated three times.

## Author Contributions

L.W.H., Z.L., F.F., W.Z.B. contributed equally to this work and should be regarded as co‐first authors. W.X.Q., D.Y.N., F.Y.H., C.H., Q.Y.J., X.H.X., and A.N., participated in performing experiments and discussed the results; L.W.H., Z.L., F.F., C.L.Y., and Z.H.C. analyzed the data and wrote the paper. F.Z., L.X., C.H.Q. and Z.M.W. reviewed and revised the manuscript. All authors read and approved the final manuscript.

## Conflicts of Interest

The authors declare no conflicts of interest.

## Supporting information




**Supporting File**: advs76971‐sup‐0001‐SuppMat.docx.

## Data Availability

The data that support the findings of this study are available from the corresponding author upon reasonable request.
