# Supplementary material for: Engineered Xenogeneic Bone Scaffold with IL‐10 Nanodelivery System: Immunomodulation and BMSC Fate Programming for Skull Defect Repair
Source: Adv Sci (Weinh). 2026 Aug 5;13(60):e76971. doi: 10.1002/advs.76971 (PMC13440199; doi:10.1002/advs.76971)
Supplement: Supplementary file 1 [file ADVS-13-e76971-s001.docx]

Supporting Information

**Engineered Xenogeneic Bone Scaffold with IL-10 Nanodelivery System: Immunomodulation and BMSC Fate Programming for Skull Defect Repair**

*Weihao Lv^1*^, Lei Zhang^1*^ Fei Fei^2*^,* *Zhibin Wu^3*^, Xiuquan Wu^1^, Ya-nan Dou^1^, Yihao Fu^1^, Hao Chang^1^, Yujie Qiang^1^, Huaxia Xie^1^, Ni An**^1^, Leiying Chen^1^, Hongchen Zhang^1^, Min-Wen Zheng ^3#^, Hongqing Chen^1#^, Xia Li^1#^, Zhou Fei^1#^*

**Figure S1. Bioactivity Assessment of Released IL-10.** (A) CD86 positive-macrophage cells proportion in different groups measured in vitro based on flow cytometry. (B, C) ELISA of IL-1β, TNFα in supernatants of RAW 264.7 in vitro in different groups. For panel A-C: **p < 0.01, ***p < 0.001, ****p < 0.0001 by one-way ANOVA analysis. Data are presented as mean ± SD, n = 3/group.

**Figure S2. Batch consistency verification.** (A-D) Quantitative residual dsDNA, α - galactose and MHC(SLA) antigen levels in different batches. For panel A-D: ns, no significance, ****p < 0.0001 by one-way ANOVA analysis. Data are presented as mean ± SD, n = 3/group.
